# Critical Assessment of the Evidence for Striped Nanoparticles

**DOI:** 10.1371/journal.pone.0108482

**Published:** 2014-11-17

**Authors:** Julian Stirling, Ioannis Lekkas, Adam Sweetman, Predrag Djuranovic, Quanmin Guo, Brian Pauw, Josef Granwehr, Raphaël Lévy, Philip Moriarty

**Affiliations:** 1 School of Physics and Astronomy, The University of Nottingham, Nottingham, United Kingdom; 2 Department of Materials Science and Engineering, Massachusetts Institute of Technology, Cambridge, MA, United States of America; 3 School of Physics and Astronomy, University of Birmingham, Birmingham, United Kingdom; 4 International Center for Young Scientists (ICYS), National Institute for Materials Science (NIMS), Tsukuba, Ibaraki, Japan; 5 Sir Peter Mansfield Magnetic Resonance Centre, School of Physics and Astronomy, The University of Nottingham, Nottingham, United Kingdom; 6 Institute of Integrative Biology, University of Liverpool, Liverpool, United Kingdom; King Abdullah University of Science and Technology, Saudi Arabia

## Abstract

There is now a significant body of literature which reports that stripes form in the ligand shell of suitably functionalised Au nanoparticles. This stripe morphology has been proposed to strongly affect the physicochemical and biochemical properties of the particles. We critique the published evidence for striped nanoparticles in detail, with a particular focus on the interpretation of scanning tunnelling microscopy (STM) data (as this is the only technique which ostensibly provides direct evidence for the presence of stripes). Through a combination of an exhaustive re-analysis of the original data, in addition to new experimental measurements of a simple control sample comprising entirely unfunctionalised particles, we show that all of the STM evidence for striped nanoparticles published to date can instead be explained by a combination of well-known instrumental artefacts, or by issues with data acquisition/analysis protocols. We also critically re-examine the evidence for the presence of ligand stripes which has been claimed to have been found from transmission electron microscopy, nuclear magnetic resonance spectroscopy, small angle neutron scattering experiments, and computer simulations. Although these data can indeed be interpreted in terms of stripe formation, we show that the reported results can alternatively be explained as arising from a combination of instrumental artefacts and inadequate data analysis techniques.

## Introduction

Scanning probe microscopy (SPM) is an exceptionally powerful technique at the core of modern nanoscience. Indeed, many would argue that the origins of the entire field of nanoscale science lie in the invention of the scanning tunnelling microscope (STM) in the early eighties [1]. Single atoms and molecules are now not only routinely resolved with STM but, under appropriate experimental conditions, can be precisely positioned [2]–[5] to form artificial nanostructures exhibiting fascinating quantum mechanical properties [6]–[8].

The development of the atomic force microscope (AFM) [9] shortly after the introduction of the STM broadened the applicability of SPM to a much wider variety of substrates — including insulators in particular — and led to the adoption of SPM as a high resolution imaging technique in very many scientific disciplines and sub-fields. The state of the art in atomic force microscopy is no longer ‘just’ atomic resolution [10] (a remarkable achievement in itself), but the imaging of intramolecular *bonds* [11]–[13] and intermolecular structure (whose origin is currently an active area of debate [14], [15]). Furthermore, SPM systems now operate in a range of environments spanning what might be termed ‘extreme’ conditions — ultrahigh vacuum, low temperatures, and high magnetic fields (for example, an STM running at 10 milliKelvin in a field of 15 T has recently been developed [16]) — to the *in vitro* application of AFM to study biochemical and biomedical processes [17]. A significant number of commercial suppliers also now provide ‘turn-key’ SPM systems such that the probe microscope has evolved into a standard characterisation tool in the vast majority of nanoscience laboratories.

Unfortunately, however, with the exceptional capabilities of the scanning probe microscope come a plethora of frustrating instrumental artefacts. These can give rise to images which, although initially appearing entirely plausible, unsettlingly arise from a variety of sources including improper settings of the microscope parameters (for example, the feedback loop gains used to control the motion of the scanning probe), external electrical or vibrational noise, and/or convolution of the sample topography with the structure of the probe. The latter is especially problematic when the features of interest at the sample surface have a radius of curvature which is comparable to that of the tip.

While some of these SPM artefacts, such as those due to improper feedback loop settings, are relatively straight-forward to diagnose and eliminate, tip-sample convolution can often require particularly careful and systematic experimental technique to identify and remove [18]. Debates in the literature regarding artefacts in atomic/molecular resolution images arising from, e.g., ‘double’ or multiple tips [19], and/or tip asymmetry [20], show that, unless appropriate experimental protocols have been used to ensure that the SPM images are as free of tip influence as possible, it can be exceptionally difficult to deconvolve the influence of the tip structure from the final image. In addition, without appropriate control samples it is entirely possible to misinterpret genuine and mundane surface features as new and hitherto unobserved aspects of the molecule or structure of interest. This latter problem was brought sharply to the fore in the early days of STM when the results of very high profile papers claiming to have attained high resolution images of DNA and other biomolecules on graphite were replicated on freshly cleaved, i.e. entirely molecule-free, substrates. The ‘molecular’ images were shown in a number of cases to arise from step edges and graphitic fragments (“flakes”) on the bare graphite surface [21].

In this paper we critique, in the context of the SPM artefacts described above, the body of highly-cited work published by Stellacci and co-workers over the last decade or so (see, for example, [22]–[26]), which claims that stripes form in the ligand shell of appropriately functionalised gold nanoparticles. These claims have subsequently led to the proposal that ligand stripes substantially influence the ability of nanoparticles to penetrate cell membranes [25], and, very recently, Cho *et al.* [26] have argued that the striped morphology enables high selectivity for heavy metal cations (although there are unresolved issues regarding the lack of appropriate control samples for this study [27]). By combining an extensive re-analysis of Stellacci *et al.*'s data with imaging of a simple control sample comprising ligand-free nanoparticles, we show that the scanning probe data published to date provide no evidence for stripe formation and instead can be explained by a combination of instrumental artefacts, data selection, and observer bias (See disclaimer at end of text). For completeness, we also consider the evidence, or lack thereof, for stripe formation from other techniques such as transmission electron microscopy [23], nuclear magnetic resonance (NMR) spectroscopy [28], small angle neutron scattering (SANS) [29], and computer simulations [30]. Taken together, our analyses provide important insights into the pitfalls of not adopting an extremely critical, systematic, and sceptical approach to SPM imaging of nanostructured samples.

## Materials and Methods

In order to demonstrate how striped features and other intraparticle structure can arise from STM artefacts, we prepared a control sample comprising entirely unfunctionalised nanoparticles. This was generated under ultrahigh vacuum conditions so as to ensure that the nanoparticle surfaces remained free of contamination and adsorbates.

Following a well-established approach [31], [32], a C_60_ monolayer (ML) was formed on the Si(111)-(7×7) surface to act as a template for the formation of Ag nanoparticles. (This strategy cannot be used to form Au nanoparticles [33], such as those studied by Stellacci *et al.* As feedback ringing and imaging artefacts are entirely independent of the composition of the nanoparticle, however, our results are equally applicable to Au nanoparticles.) C_60_ was first sublimed onto a clean Si(111)-(7×7) surface, formed using standard flash annealing procedures [34]. Following the deposition of a multilayer fullerene film, the sample was annealed at ∼450°C to desorb all C_60_ other than the first chemisorbed monolayer. Silver was then deposited from a Knudsen cell operating at a temperature of approximately 880°C onto the 1 ML C_60_/Si(111) sample. In order to modify the size distribution of the Ag nanoparticles — so as to make the particles' mean diameter comparable to that of those studied by Stellacci *et al.* — we subsequently annealed the Ag-covered C_60_ monolayer sample in the 200°C to 400°C range.

Our STM measurements were acquired using an Omicron Nanotechnology low temperature ultrahigh vacuum qPlus atomic force microscope–scanning tunnelling microscope instrument operating at 77 K at a pressure of ∼ 5×10^−11^ mbar. All SPM image analysis in this paper is performed using scripts written in MATLAB using the SPIW toolbox [35]. The raw data and scripts have been made public [36] to allow our analysis to be repeated and/or modified by any interested party.

## Results and Discussion

In the following sections we re-analyse the evidence for striped nanoparticles that has been presented by Stellacci and co-workers in a series of papers over the last decade. Where necessary, we complement the re-analysis of Stellacci *et al.*'s data with a discussion of STM measurements of the Ag nanoparticle sample described in the preceding section. A key advantage of the protocol we have adopted for nanoparticle synthesis is that the Ag particle surfaces in our experimental measurements are entirely ligand free. As such, they act as excellent control samples to highlight the role of instrumental artefacts and improper data acquisition/analysis protocols when making claims for structure in a ligand shell.

Following criticism of the evidence for stripes by Cesbron *et al.* [37], some raw STM data from the first papers published by Stellacci *et al.* [23], [24], [38] was placed in the public domain [39]. For reasons detailed in the following sections, the archived data do not, however, justify the conclusions drawn in these papers. A number of other papers based on STM data have also been published since the archived data was released [29], [40], [41] and we are grateful to the corresponding author of one of those papers [41] for providing some of the data associated with that work for re-analysis. We examine and provide a detailed critique of these STM data, and we discuss the evidence, or lack thereof, for stripe formation from a variety of other techniques.

### Striped features in SPM images arising from feedback instabilities

**Figure 1:**
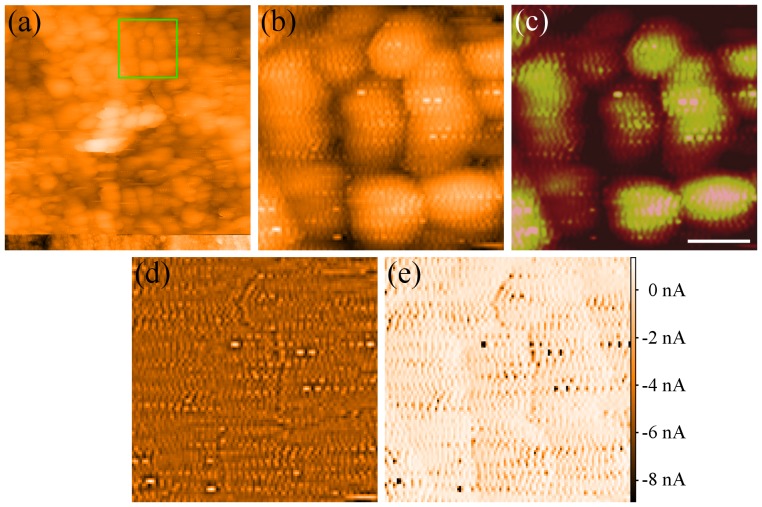
Reanalysis of the data for Figure 1a) of Jackson et al. 2004 [23]. (a) The raw 157nm wide image collected for Jackson et al. (b) Zoom-in on a 37 nm wide area marked in green on(a). This image has been flattened using first order plane subtraction. It is clear that the ripples extend between the particles. (c) Figure 1a) from Jackson et al.(2004) Spontaneous assembly of subnanometre-ordered domains in the ligand shell of monolayer-protected nanoparticles. Nature materials 3: 330–6. (DOI:10.1038/nmat1116) Reproduced by permission of Nature Publishing Group. Note that the choice of contrast obscures the ripples between the particles. Scale bar 10nm. (d) Fourier transform high-pass filter of (b), removing spatial frequencies below 0.33 × 109 m−1. (e) Simultaneous current image of (b). Note the (inverted) similarity to (d). The colour ranges for (a), (b) and (d) are set to run linearly from the highest to the lowest pixel. For (e) the colour range is set to run linearly for the centre 99.6% of pixels, as extreme pixels mask much of the contrast (For this section of the image the tunnel current spans a range from -51.2 nA to 2.83 nA). Colour bar shows recorded current values, the setpoint current is +838 pA.

The first paper on the striped morphology of Au nanoparticles (Jackson *et al.* 2004 [23]), leads with an STM image of nanoparticles having a mixed 1-octanethiol (OT) and mercaptopropionic acid (MPA) termination, showing striped features on each nanoparticle (reproduced in Figure 1c below). This is one of the clearest STM images of stripes of which we are aware and played a seminal role in establishing the concept of “striped” ligand patterns on Au nanoparticles. Before we discuss the compelling evidence that the stripes simply arise from a well-known STM artefact, and not from ligand organisation, we first note that the contrast in the image is saturated at the lower end of the contrast scale (i.e black). If we instead set a linear contrast scale from the highest to lowest pixel (as is standard practice) it is clear that *the stripes extend between the nanoparticles* (Figure 1b). This observation alone strongly suggests that the stripes are not real surface features confined to the nanoparticles. Equally problematic, however, is the alignment of the ‘stripes’ with the slow scan direction. It is physically impossible for the ligands on the variably oriented surfaces/facets of the gold nanoparticles to spontaneously align in this fashion. (See also our discussion of Yu and Stellacci [42] in the “Pixellation, offline zooms, and interpolation” section below).

We note in passing that the image from Jackson *et al.* 2004 [23] included as Figure 1c) is a 38×38 nm^2^ offline zoom of a 157×157 nm^2^ image (Figure 1a). To increase the apparent resolution the image was interpolated up to a much larger number of pixels by Jackson *et al.*, and possibly filtered to give rounded shapes to features which are only 2–3 pixels across. We will return to a discussion of how this type of image processing can give rise to misleading results.

To understand scanning probe microscopy image artefacts it is first necessary to realise that the images are formed by bringing a sharp tip close to the surface under study. In the case of STM, a feedback loop controlling the tip-sample separation is used to maintain a constant tunnelling current between the tip and the surface. By recording the 3D path taken by the tip as it is raster-scanned over the surface, a height profile is taken. Improper choice of scan speed or feedback gains can result in poor regulation of tunnel current or even complicated feedback instabilities. In addition, as the current to be regulated is of the order of nanoamps, the effect of electrical noise cannot be neglected. Furthermore, even assuming perfect feedback conditions, the image is a convolution of the surface and tip structure, combined with the presence of tip-sample forces, which can cause changes to either (or both) during image acquisition, resulting in abrupt modifications.

Thus, to reliably verify the existence of specific topographic structure it is important to systematically probe the features by comparing the trace and retrace images from the STM, taking repeat scans of the same feature, rotating the scan direction, deliberately modifying the tip in order to ascertain the level of tip-sample convolution, and zooming in on specific features in ‘real time’, i.e. by reducing the scan area imaged by the STM, to check that features are unchanged [43]. Based on these observations it would appear that these basic checks on image consistency were not carried out in the original, highly cited, Jackson *et al.* 2004 [23] paper, nor in much of the subsequent work on striped nanoparticles.

To help the user identify artefacts arising from improper feedback settings, scanning probe microscopes normally also save images from other data channels in addition to the topography channel. An important diagnostic tool is the error signal (or current image), i.e. the difference between the setpoint value and the measured current. Ideally, the current image should be blank, but as the feedback is not instantaneous there is normally some surface structure visible. Strong, clear features in the tunnel current image, however, imply the feedback is not performing correctly. More importantly, the values of the pixels in the tunnel current image should not differ dramatically from the setpoint current used to acquire the topographic image.

Figure 1e shows the tunnel current image recorded simultaneously with Figure 1b. The structure from the topography image is clearly visible in the tunnel current map. It is possible to remove the curvature of the nanoparticles from the topography using a Fourier transform approach to filter out spatial frequencies below 0.33×10^9^ m^−1^ (Figure 1d). This further enhances the similarity to the tunnel current image, strongly suggesting that any sub-nanoparticle resolution results solely from tunnel current tracking errors. The full code used to generate Figure 1 is presented in the public data archive [36].

There are, however, even more fundamental problems with the tunnel current image shown in Figure 1e). From the data archive placed in the public domain by Stellacci *et al.* [44], we find that the image was taken with a current setpoint of 838 pA and a sample bias of 1V (despite the text of the paper stating the images were recorded with setpoints of 500–700 pA). Pixel values from the (full) current image range from 20.2 nA to -98.2 nA. These values are clearly unphysical as the current changes sign while the voltage does not. The tunnel current values have been confirmed in Gwiddion [45], WSxM [46], and NanoScope Ver 5.31r (the software used to record the original image). It is important to note that programs such as WSxM and NanoScope automatically pre-process images by background subtracting or truncating the *z*-range. Such pre-processing must be turned off to restore the correct current values.

A possible explanation for these results, as suggested by the Stellacci group [47], is that their microscope was set to automatically background subtract the tunnel current data before saving, and thus the raw images were never correctly saved. The implications of this are that the true current range, which should be largely unaffected by background subtraction, is of order 118 nA. If we apply the most fair attempt at inverting this subtraction by shifting all pixel values until the lowest point reaches zero this would give a mean tunnel current of order 98 nA, orders of magnitude above the setpoint. This is far above the normal range of currents expected for accurate STM measurements of nanoparticle assemblies.

Another explanation is that the current-to-voltage amplifier saturates to a value of -100 nA for any currents outside its measurement range of ±100 nA. Negative pixels result from averaging of positive signals with −100 nA during saturation. This explanation would imply that the current preamp was regularly saturating to over 100 nA while feedback tries to maintain a setpoint of less than 1 nA.

The key point is that, regardless of which explanation for the negative current values is correct, the tunnel current image clearly exhibits exceptionally strong oscillations in the error signal. These arise from improper setting of the feedback loop gains (and other scan parameters). It is thus feedback loop oscillation, and not the self-assembly of two different ligand types, which gives rise to the stripes observed in the STM images shown in Jackson *et al.* 2004 [23].

**Figure 2:**
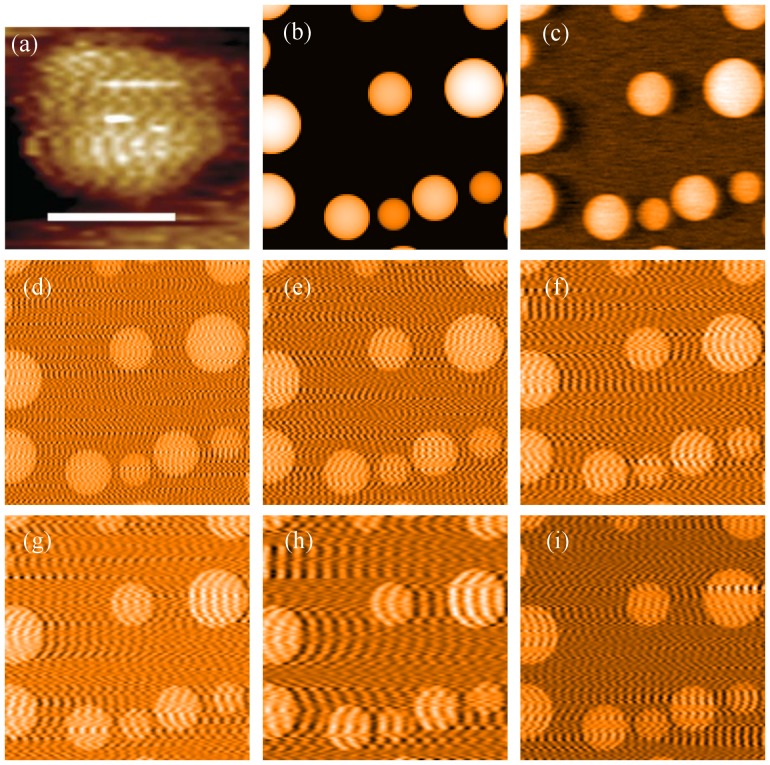
Comparison of STM image of nanoparticle “stripes” with simulated STM feedback results. (a) Image from [48] Uzun et al. (2008) Water-soluble amphiphilic gold nanoparticles with structured ligand shells. Chemical communications (Cambridge, England): 196–8. (DOI: 10.1039/B713143G [48]), reproduced by permission of The Royal Society of Chemistry. The image shows features which can be reproduced by simulated SPM feedback (scale bar 5 nm). (b) Surface topography used in all numerical simulations. (c) Numerically simulated image with appropriate parameters Kp = 500 and Ki = 100. (d–h)The same simulation with Kp = 50 and Ki = 8000, 5000, 3000, 2000, and 1000 respectively. Image (i) is the retrace image recorded while recording image (f) presented directly above.

Further images produced by Stellacci *et al.* show very similar contrast to those included in Jackson *et al.* 2004 [23], including, for example, Figure 2a), reproduced from Uzun *et al.* [48]. To assess whether these images also arise from feedback artefacts, and without having access to the raw data, we have used simulated SPM feedback to generate expected images from improper feedback settings, as shown in Figures 2c–i). Before giving details of the simulation we present a brief summary of how feedback is implemented in a real STM.

STM feedback utilises a proportional-integral (PI) controller feedback mechanism, similar to the common proportional-integral-derivative controller, but without the derivative component, as this acts as a high pass filter amplifying noise. The proportional part of this controller simply records the error signal (the difference between the setpoint amplitude and the recorded amplitude), multiplies this by a gain factor (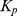), and adds this to the extension of the piezo. The integral part of the controller integrates the error signal over time and multiplies by a separate gain (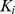). This removes steady state errors which arise from effects such as sample drift and cannot be corrected using simply a proportional controller. The trade off with adding the integral controller is that the tip position overshoots the optimal position before returning. If 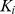 is too large the feedback can become unstable and oscillate about the optimal position. Therefore, for stable imaging it is necessary to carefully adjust 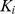 and 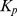 in order to reduce the error signal.

A real STM controller performs all measurements at discrete time intervals and does all calculations numerically. As such, we have written a numerical simulation, which mimics the STM's response to a given topography, by implementing a PI controller (Figure 2b)). For this simulation each measurement is subject to white noise to simulate electrical noise. Full details of the simulation, and all code used, are provided in the File S1 and the public data archive [36]. Analytical methods for this type of control theory modelling are available and have very recently been used by Stellacci and co-workers [41]. We stress, however, that the method adopted by Stellacci *et al.* [41] inadvertently produces oscillations arising from incorrect modelling of mechanical components and the PID loop itself, rather than from feedback instabilities [49].

Using the simulation methods described in the File S1, it is straightforward to generate images of a smooth surface which appear to show stripes (Figure 2d–h)) by choosing an inappropriately high integral gain coupled with a low proportional gain. As the integral gain is increased the width of the stripes can be modified. Using more appropriate imaging parameters (Figure 2c)) instead, the surface structure can be accurately reproduced. It is also important to note that when the trace and retrace images — recorded when the tip is rastering in opposite directions — are compared, the curvature of the stripes changes (Figure 2f and i). This difference between trace and retrace images is a common method used to identify feedback instabilities but, unfortunately, until only very recently was not used by Stellacci *et al.*

To complement the results of the simulations we have grown Ag nanoparticles using the procedure described in Materials and Methods, and imaged these particles under various gain conditions with an Omicron low temperature STM. One minor disadvantage of using the Omicron microscope in the standard software configuration for this test is that the proportional and integral gains cannot be varied separately using the control software. Instead, a combined feedback gain is set as a percentage of the maximum allowed gain.

**Figure 3:**
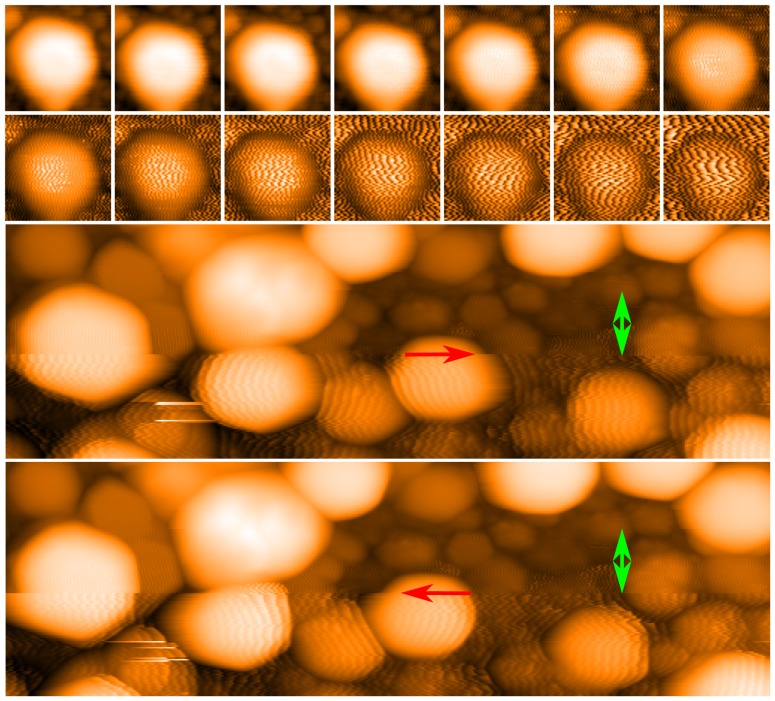
Imaging of unfunctionalised Ag nanoparticles with varying scan parameters. Top two rows: The top left image was recorded with a gain of 5%. For each consecutive image (i.e. moving along the rows from left to right), the gain was incremented by 1%. Each image is 8 nm wide, and all were recorded with a tip speed of 38 nm/s. Bottom two rows: Trace (third row) and retrace (bottom row) image of Ag nanoparticles, upwards scan direction. At the point marked by an arrow in both images, the scan speed was reduced from 514 nm/s to 195 nm/s, causing a significant reduction in stripe width (indicated with red arrows; these arrows also indicate scan direction). Soon after, the gain was reduced from 22% to 10% and the stripes disappear (gradually decreased in the lines marked by the green double-headed arrow). Both images have a width of 50 nm.

Figure 3 shows consecutive images of the same nanoparticle taken at increasing gains. In agreement with the simulations shown in Figure 2, at an appropriate gain setting the STM image shows the bare featureless nanoparticle surface. As the gain is increased, striped features appear in the STM image. In addition, and as predicted by the simulation, the stripes vary in both contrast and width as the gain is increased. As the frequency of feedback oscillations is dependent on both the proportional and integral gain, which, in our case, are not known separately, we cannot directly compare the evolution of stripes in the experiment with those in the simulation (where the proportional gain is constant). Nonetheless, the key conclusion is clear: improper feedback loop settings produce stripes whose spacing depends on the loop gain. All images represent a 71 pixel × 71 pixel section of a 512 pixel × 512 pixel image, which was then bi-cubically interpolated up to 284 pixels × 284 pixels to mimic the interpolation in published STM images of “striped” nanoparticles.

### Assessing the statistical analysis used to distinguish artefacts from real structure

Notwithstanding the discussion in the previous section, Stellacci *et al.* have argued that they can distinguish between feedback loop artefacts and true nanoparticle topography. In two publications [24], [38] following the Jackson *et al.* 2004 [23] paper critiqued above, a statistical analysis of previous STM data (from their group) was used to suggest that feedback artefacts could be differentiated from real topographical structure. In this section we critically consider the evidence for that claim. Before doing so, it is perhaps worth noting that an experimental protocol which involves setting abnormally high loop gains to distinguish between “real” stripes and those due to high loop gains is not a particularly robust approach to making STM measurements. A rather more compelling strategy would be to ensure that the loop gains were set appropriately and to demonstrate that, under conditions where the tip is accurately tracking the surface, stripes similar to those shown in Jackson *et al.* 2004 [23] remain visible. Throughout all of the work published by Stellacci *et al.* this has not been achieved. We return to this point repeatedly below.

The key claim of Jackson *et al.* 2006 [24] is that it is possible to distinguish between noise and ripples arising from real nanoparticle structure. In Figure 3 of that paper [24] changes in noise and ripple spacing as a function of tip speed are shown. The caption for that figure states that “*Each point in the plots is the average of multiple measurement*s”. This is highly misleading, however, as only one image, *of a different surface area each time*, was taken for each tip speed. The multiple “measurements” are, therefore, simply multiple readings of spacings of different features in the same image, and *not of the same particle*.

The spacings described in Jackson *et al.* 2006 [24] were determined by measuring the separation between high intensity pixels in the images — which, again, are interpolated zooms of larger area scans — and are quoted in the image annotation to a rather optimistic significance of 10 pm (It is worth noting that 10 pm equates to a separation of 0.026 pixels in the raw, uninterpolated image). The distances measured range from approximately 2 to 4 pixels and thus are very close to the (Nyquist) resolution limit of a 2 pixel spacing. We note that this combination of large area scanning followed by highly interpolated offline zooms is a rather unorthodox approach to scanning probe microscopy that, for good reason, is not widely applied within the SPM community.

**Figure 4:**
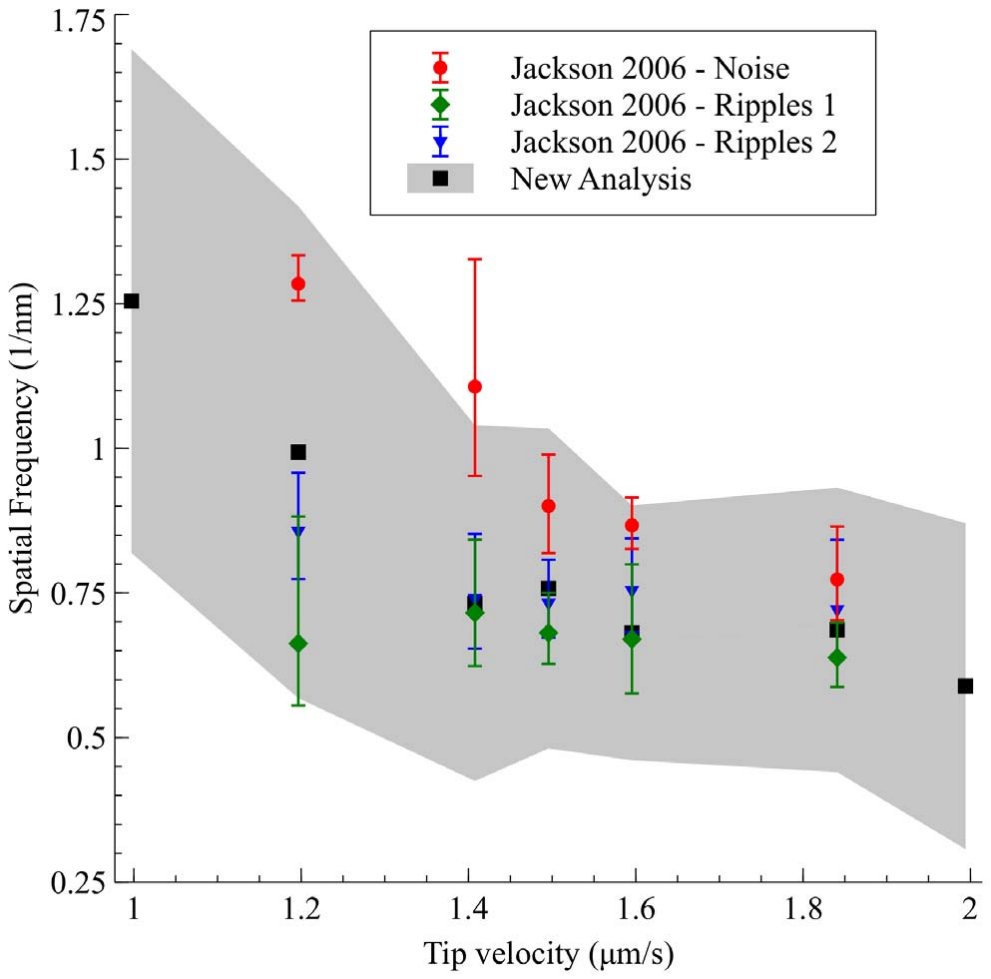
Reanalysis of the data for **Figure 3** of Jackson et al. 2006 [24]. The black squares represent the peak frequency in the Fourier spectrum of the tunnel current images, while the grey area represents the full width at half-maximum (FWHM) of the peak in Fourier space. Red circles are digitised data from the noise spacings presented in Figure 3b of Jackson et al. 2006 [24]. Green diamonds and blue triangles are digitised data from the ripple spacings presented in Figure 3(b) of Jackson et al. 2006. All ripple spacings fall inside the spatial frequency band of the error signal. The first and last point represent images archived by Stellacci et al. along with the data for Figure 3 of Jackson et al. 2006 [24], but which were not analysed in Jackson et al. 2006. The full method and code used to generate this figure are given in the Supplementary Information.

To put the analysis of the feedback noise contributions on a much sounder quantitative footing, we have performed Fourier transforms of the fast scan lines of the tunnel current images associated with Figure 3 of Jackson *et al.* 2006 [24], as feedback noise should dominate in the current channel. Feedback noise will also be aligned along the fast scan direction. We then combined the power spectra from each of the scan lines to locate the peak spatial frequency and the full-width at half maximum (FWHM) of the peak in the Fourier spectrum. The FWHM of the spectral peak gives a good measure of the range of frequencies which can arise from feedback noise. Plotting these spatial frequencies along with digitised data from Figure 3 Jackson *et al.* 2006 [24], as shown in Figure 4, it is possible to show that *all* of the quoted ripple spacings fall within the broad background noise measured for the whole image, and are hence not significant. One should also note in Figure 4 the systematic overestimation of the noise spatial frequency and underestimation of the noise error bars in the analysis by Jackson *et al.* 2006 [24], further demonstrating the inaccuracy of measuring ripple spacings by counting relatively few pixels.

Jackson *et al.* 2006 [24] also state that the gold foil substrates used in the work have “*curvature comparable to that of the nanoparticle core*”. This begs the question as to just how some areas were objectively defined as the surface, and thus exhibited feedback noise, while others were defined as nanoparticles with molecular resolution. Furthermore, the areas defined as nanoparticles in the images do not show clear striped domains. Instead, they show a disordered noisy pattern.

For all of these reasons, the conclusions drawn by Jackson *et al.* 2006 [24] regarding their ability to distinguish true topographic “stripes” from feedback loop artefacts are entirely unreliable. Before we move away from the discussion of Jackson *et al.* 2006 [24], we would like to bring to the reader's attention to the processing of images. The selected scale of Figure 4 of the paper shows only very few levels of contrast, as such, the image appears as more of a contour map than a real STM image. Equally striking is Figure 9b of Jackson *et al.* 2006 [24]. In the context of discussing the orientation of stripes while rotating the scan angle, the inset, which is referred to as “Enlarged image of the same nanoparticle as in (a)”, is actually an angled 3D rendering of the image, thus distorting the scan angle and providing an unfair comparison. Figure 8d of Jackson *et al.* 2006 [24] has lines drawn to “guide the reader's eye” to the direction of the stripes, arguing they are not aligned to the scan direction. This, however, masks the contrast and is yet again very misleading. An examination of the region which was enlarged simply does not show clear stripes in this direction.

We now turn to the second paper from Stellacci and co-workers which “critically assessed” the STM evidence for the striped morphologies: Hu *et al.* [38]. This paper solely concentrated on statistical analyses of their STM data. In common with Jackson *et al.* 2006 [24], the central claim is the ability to differentiate between stripes formed from feedback noise and those arising from real topographic features. This was ostensibly based on a “rigorous” statistical analysis, where ripple spacings — again measured by eye, and thus subject to the same observer bias present for the analysis in Jackson *et al.* 2006 (Figure 3) — were compared to noise spacings while the tip speed was changed.

In one aspect the methodology is improved from that in Jackson *et al.* 2006 [24], in that separate images were used for topographical ripples and noise. The experimental methodology nonetheless still suffers from various other fundamental flaws. For a rigorous comparison, as the authors claim, each image taken at varying tip speeds should be of the same sample area, with the same scan size, and with the same feedback gain settings. The gain settings are especially important as we have shown above that the ripple spacing depends on feedback gains as well as tip speed. The archived data provided for the Hu *et al.* [38] paper has a selection of non-consecutive images, with sizes ranging from 2 to 300 nm, each with different gains, of different areas of the sample, or often of entirely different samples. As so many experimental variables are changing it is impossible to isolate the effect of tip speed, especially as gains have a pronounced effect on stripe width (Figures 2 and 3).

We also disagree with the ambiguous descriptions of data acquisition in Hu *et al.* [38]. When describing the influence of tip speed on ripple spacings it is stated that “Many images are analyzed at varying tip speeds. In some cases we have analyzed as many as 10 images”. Originally we understood this to mean that each speed had as many as 10 images, and the resulting data point was an average. After receiving the archived data (along with private communications with the research group [47]) we have found that each data point (i.e. for a given tip speed) is instead from a single image. The “10 images” refers simply to ten separate data points, each with different speeds, taken on different areas of the same sample (with other changing experimental conditions). Furthermore, the number of data points, indicated for different samples, does not agree with the number of images provided: at times the archive is missing images, and for other samples, more images are provided than were measured.

### Pixelation, offline zooms, and interpolation

Cesbron *et al.* [37] identified that the striped features observed for mixed-ligand-terminated particles, as of 2012, were all aligned with the scan direction. This was used as a central argument of the paper to suggest that the stripes were not true features but artefacts from feedback loop ringing (The analysis of the raw data described above confirms this interpretation). In response to Cesbron *et al.*'s criticism, Yu and Stellacci [42] provided examples of stripes which apparently were not aligned with the scan direction. Those particular images, however, while not exhibiting feedback loop instabilities, suffer from a combination of poor experimental design, flawed analysis techniques, and strong observer bias, which we also critique in depth in the following.

The images in Figures 3 and 4 of Yu and Stellacci [42] were recorded using an Omicron micro-STM under UHV conditions, a microscope capable of acquiring high resolution images of just a few nm across, and of providing atomic resolution on flat surfaces [50]. The images, however, were acquired using a scan area of 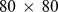 nm^2^ (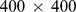 pixels), on nanoparticles with a diameter of order 4–6 nm. No data were presented where the scan range was decreased to record high-resolution images. Instead, zooms were yet again performed offline. Yu and Stellacci presented further enlarged figures showing single nanoparticles which were of order 30 pixels across, with a particle itself having a diameter of order 20–30 pixels. These images were then (inadvertently) interpolated via an image analysis package to show smooth “stripe” features. The “stripes”, however, arise from as few as 2–3 noisy pixels in the original, uninterpolated, image. As such, this is a fascinating example of how improper image acquisition and analysis, coupled with observer bias, can lead to the observation of features which do not exist.

The human brain is well known to recognise expected patterns were none are present [51], [52]. A particularly important example is the observation of *perceived* correlated features in Poisson point distributions (where no spatial correlation exists). To ascertain whether stripes are present, therefore, it is important to carry out a rigorous quantitative analysis. Although, to the very best of our knowledge, no high resolution images were ever taken by Yu and Stellacci, many low resolution images of the same sample area were acquired (which the corresponding author kindly sent to us for analysis). These repeated images of the same sample area can be used to demonstrate that the stripes, which are claimed to be present in Figure 3 and 4 of Yu and Stellacci [42], arise from a misinterpretation of random noise.

**Figure 5:**
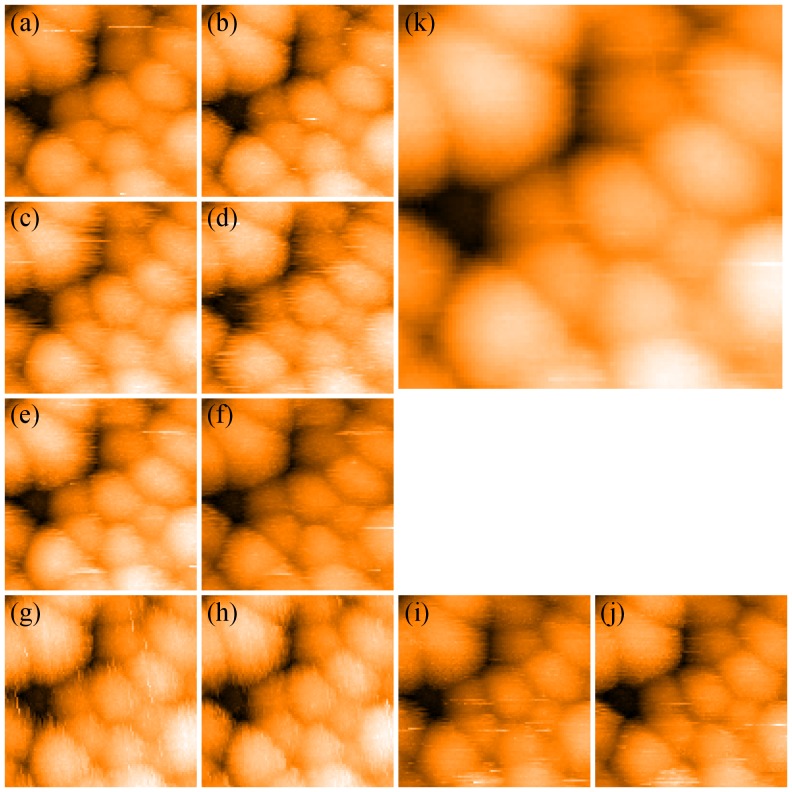
Arithmetic addition of images from Yu and Stellacci [42]. (a)-(j) Images of the same set of nanoparticles taken from each of the five trace and five retrace images provided by Yu and Stellacci. (a,c,e,g,i) are the trace images, while (b,d,f,h,j), respectively, are the corresponding retrace images. (k) Arithmetic addition of all 10 images. Note that the particles in the summed image appear entirely smooth, indicating that the features designated as stripes by Yu and Stellacci arise from noise and not real topographic structure on the nanoparticles. All images are 20 nm wide.

First, we note that the ‘full’ images in Figure 3 of Yu and Stellacci are digital zooms (∼40×40 nm^2^) of the original 80×80 nm^2^ images. A cursory analysis shows that the original images shift only by 4–5 nm between scans. Thus, it would have been easy for the authors to locate precisely the same particles and show that, if the features did indeed arise from organisation in the particle ligand shell, the stripes for all of the particles remained unchanged as the scan speed varied. This is not what is included in the paper (for reasons which will become clear). Instead, for each scan included in Figure 3 of Yu and Stellaci [42], the selected nanoparticles are different. This selection suggests consistency between the images when none is present. To highlight this, we show in Figure 5 the summation of a 100×100 pixel section of all five images from both Figures 3 and 4 of Yu and Stellacci (trace and retrace, in total a sum of ten images), where these images have been aligned using cross-correlation. If the stripes identified by Yu and Stellacci [42] arise from a source other than noise they should still be visible in the sum of the images (The summation of data in this manner is a basic protocol in experimental science to increase signal-to-noise ratio). The summed data, however, shows smooth particles and the inescapable conclusion is that the stripe features arise solely from noise.

Yu and Stellacci used the same set of images to suggest that identical features can be recognised after a scan rotation. First, if features are supposedly visible in consecutive images after a rotation, it cannot simultaneously be argued that the ligands (or particles) shift sufficiently from scan to scan such that the stripes cannot be resolved in consecutive images. Let us assume, however, that we adopt the argument, entirely lacking in self-consistency, that features on the same particle which rotate as a function of scan rotation somehow are not present from scan to scan. Those features should nonetheless be present in the retrace image, which is taken *at the same time as the trace image*.

**Figure 6:**
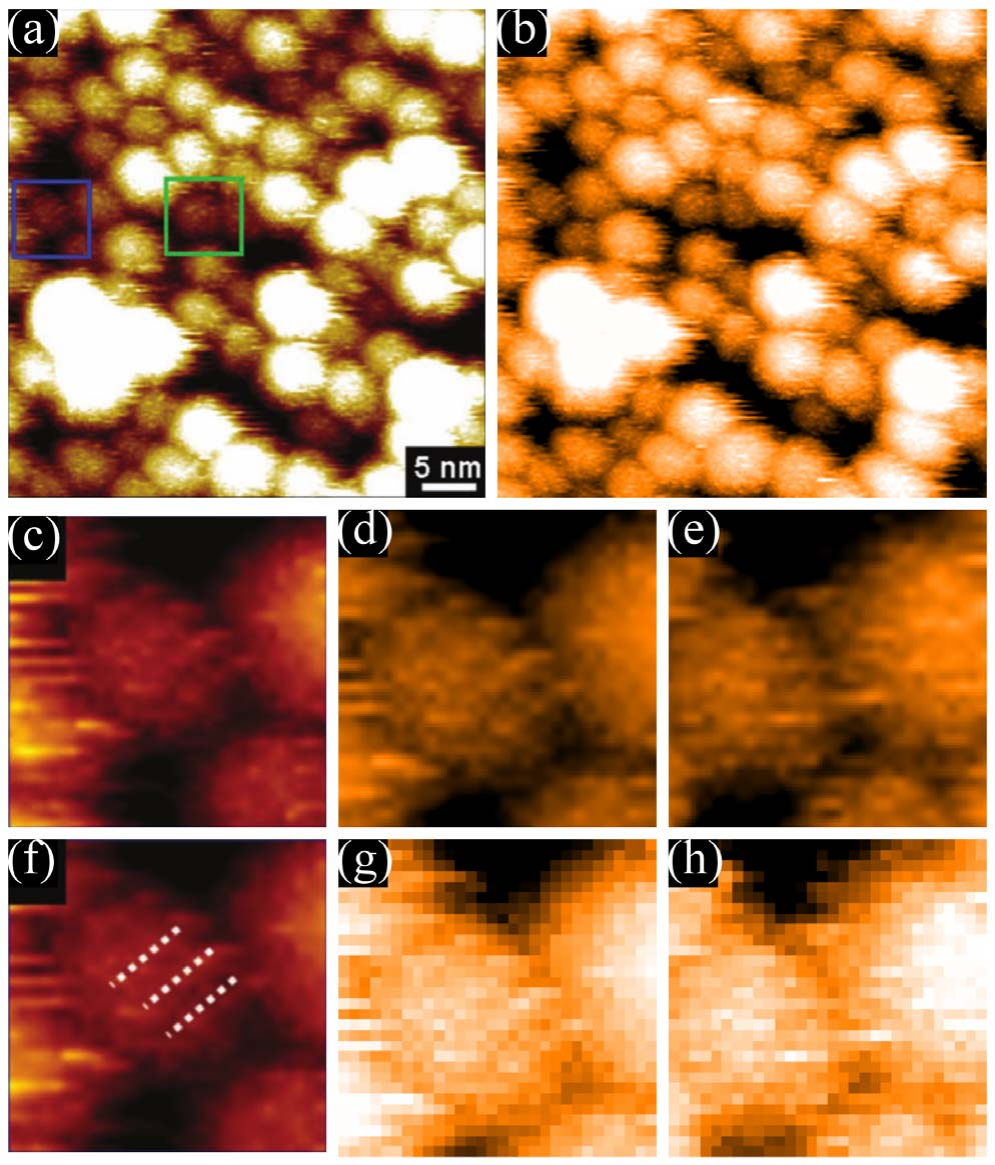
Reanalysis of data from Yu and Stellacci [42]. Panels a, c and f reproduce images from Yu and Stellacci (2012) Response to stripy nanoparticles revisited. Small 8: 3720–3726 (DOI: 10.1002/smll.201202322) - reproduced by permission of John Wiley & Sons. (a) Image as presented in Yu and Stellacci (b) A 205 × 205 pixel section of the raw data which has been processed with second order background subtraction, the colour range reduced to just 40% of the original range, and the number of pixels interpolated to best match the image shown in (a); (c) Enlargement of region highlighted by a blue square in (a); (d) Zoom of a section of the image shown in (b) taken after interpolation and colour saturation; (e) Retrace image acquired simultaneously with (d); (f) Image shown in (c) but with the stripes identified by Yu and Stellacci highlighted using dashed lines; (g) Uninterpolated zoom of the raw data showing the true pixelation. (h) Retrace image acquired simultaneously with (g). The “stripes” in (f) not only arise from a very small number of fortuitously aligned pixels, but they are not present in the retrace images shown in (e) and (h).

Figure 6a), (c), and (f) show images from Yu and Stellacci with arguably the strongest contrast of all of the features presented in that paper. Figure 6b) is a 205×205 pixel section of the raw data. In order to recreate the contrast in (a) we have flattened with a second order polynomial and then over-saturated the image by running the colour range from 35% to 75% of the full data range, before finally interpolating up to 820 pixels. Figure 6d) shows a crop of Figure 6b) showing approximately the same area as in (c), whereas (g) is the raw uninterpolated image where the individual pixels may be discerned. (The colour range is again reduced to increase contrast). Figure 6e) and (h) are equivalent to Figure 6d) and (g) respectively taken for the simultaneous retrace where we note that the stripes are not present on this image. We again must conclude that the “stripes” identified by Yu and Stellacci arise purely from a combination of noise and strong observer bias. In the public data archive [36] a program is included which allows the user to browse the trace and retrace images from Yu and Stellacci [42] (both raw and interpolated) simultaneously to show that this result is consistent across all particles and all images.

### The state of the art in resolving “stripes” — Data published in 2013

Three further papers claiming to have found evidence for stripes in STM images have been published in 2013. We start with a consideration of Ong *et al.* [40]. This work details new data acquired by three separate STM groups (including that of Stellacci) from the same samples [53]. The images collected are certainly of significantly higher resolution and of higher quality than images presented in earlier work. Despite this increased resolution, however, there is a pronounced absence of stripes in the images presented by Ong *et al.* [40].

**Figure 7:**
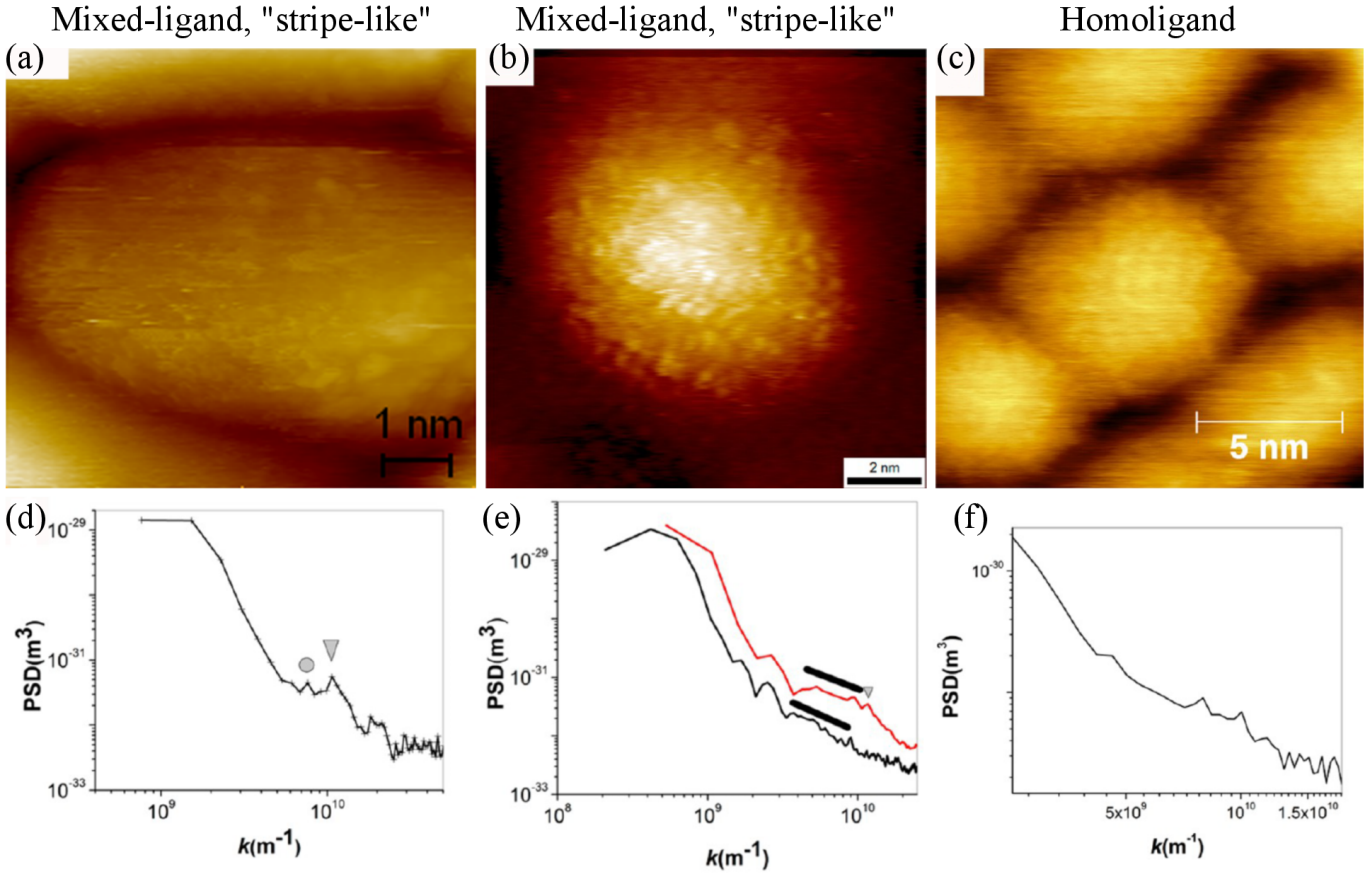
Representative data from Ong et al. [40]. Reproduced from Ong et al. (2013) High-resolution scanning tunneling microscopy characterization of mixed monolayer protected gold nanoparticles. ACS nano 7: 8529–39 (DOI: 10.1021/nn402414b) - reproduced by permission of the American Chemical Society (a) High-resolution STM image of an Au nanoparticle with a coating of 11-mercapto-1-undecanol and 4-mercapto-1-butanol, taken in UHV conditions at 77K. (b) High-resolution STM image of an Au nanoparticle with a coating of OT:MPA used in the original striped morphology paper (Jackson et al. 2004 [23]). (c) High-resolution STM image of homoligand nanoparticle with an OT coating. (a) and (b) allegedly show stripe-like domains while (c) does not. (d–f) Radially averaged PSDs from STM images of the same type of particles shown in (a)–(c) respectively.

It is particularly instructive to compare the high contrast stripes presented in Figure 1a) with the STM images of mixed-ligand nanoparticles acquired by Ong *et al.*, which are shown in Figure 7a) and (b). These latter images reputedly show individual ligand head groups arranged in stripe-like domains. For further comparison, Figure 7c) shows an image of a homoligand nanoparticle from the same paper; stripes are supposed to be absent from homoligand particles.

Ong *et al.* [40] use the persistence of features in trace and retrace images, and in consecutive images, as evidence that the features in the images are real. It is worth noting that we used precisely this approach in the preceding section to show that the stripes in the STM images of Yu and Stellacci [42], published less than a year before Ong *et al.*'s work, are clearly artefactual. However, the persistence of features from scan to scan in the data shown in Ong *et al.* is somewhat irrelevant: the scanning protocol provides no support for the presence of a striped morphology in the shell of mixed-ligand terminated particles, because the evidence for the presence of stripes in the STM data is far from compelling. Nonetheless, the data of Ong *et al.* [40] highlight an important misconception in the analysis of SPM images which we feel needs addressing before we critique that paper in detail.

The difference between trace, retrace, and subsequent images is useful to identify feedback artefacts and noise-induced features. However, this approach simply cannot identify artefacts produced from tip-sample convolution. If the tip has a similar radius of curvature to features on the surface then convolution can be very pronounced [54]. This can even be used to produce images of a tip instead of the sample [34], [55]–[57]. For this reason, the ‘internal’ contrast of nanoparticles must be considered in the context of the apparent structure of neighbouring particles (or other surface features). Note that Figure 7b), for example (and unlike Figure 7c)), shows an isolated particle with no surrounding nanoparticles with which to compare the internal structure.

**Figure 8:**
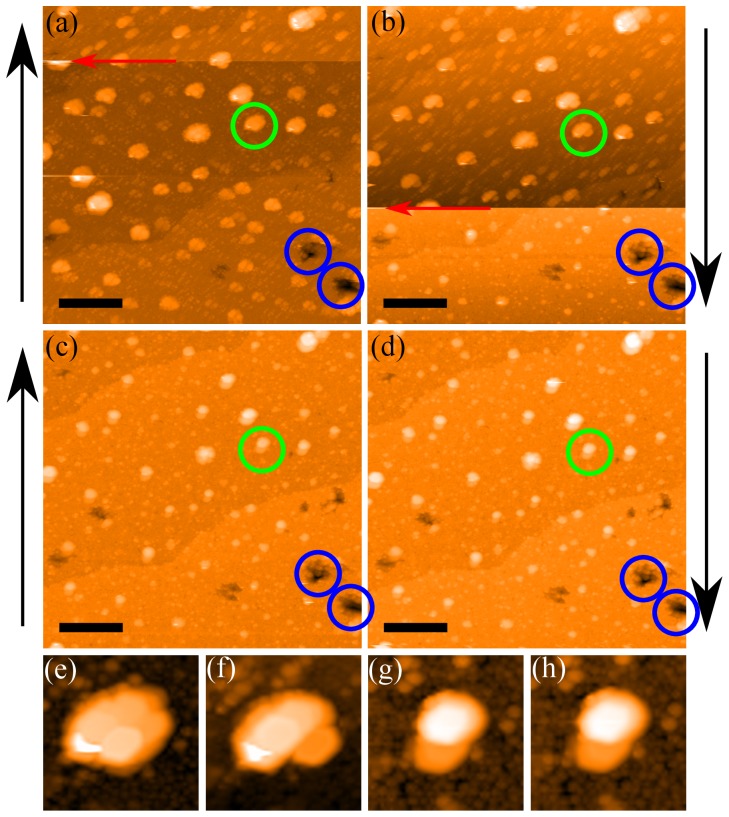
The persistence of tip induced features on bare Ag nanoparticles. Four successive images (a–d) with black arrows showing the direction of the slow scan. Tip change events, marked by a red arrow, change the apparent sub-particle structure of the bare nanoparticles. Note the persistence of the artefacts throughout the images. The tip state shown in (d) was persistent over many consecutive scans. The green circle identifies the same particle in subsequent images and (e–h) show offline (and interpolated) zooms of this particle from each of the images (a–d). Blue circles mark the same features in all images as a reference point to show the scan area is consistent. All scale bars in (a–d) are 30 nm. Minor contrast adjustment has been applied to images (a,b,d,e,f).

To highlight the influence of the tip state on the apparent structure of nanoparticles, Figure 8 shows a series of images of the Ag nanoparticle sample, which was used for the loop-gain dependent studies shown in Figure 3. Each particle clearly exhibits detailed internal structure *which is entirely artefactual* and which, although being of the same general form across the image, varies somewhat in detail from particle to particle due to changes in nanoparticle structure, and thus the nature of the tip-sample convolution. In the row of images at the bottom of the figure we show how the apparent topography of just one of the nanoparticles varies as a function of the tip structure. There are a number of tip change events (red arrow) throughout the sequence shown in Figure 8, but it is important to note that during the intervals between the tip changes the images are entirely stable and checks of image “integrity” such as rotating the scan angle would show that the particle sub-structure behaved as one would expect real structure to behave. It is also interesting to note from Figure 8 that “Janus” nanoparticle [58] artefacts are very commonly produced in STM images due to tip structure (see, for example, the lower half of Figure 8a) and both Figure 8c) and (d)). The alignment of the features identified as Janus nanoparticles in the STM images of Kim *et al.* [58] could perhaps be alternatively interpreted in terms of a directed self-assembly mechanism arising from the structure of the ligand shell of the particles. Nonetheless, the presence of a tip artefact, such as that exemplified by Figure 8, is a simpler and arguably more compelling explanation. There is also, of course, the possibility that the isolated “Janus” structures in STM images (e.g. those in Ong *et al.* [40]) arise not from the ligand shell but from paired particles (due, for example, to sintering arising from variations in ligand concentration on the nanoparticle surfaces).

Returning to the discussion of Ong *et al.* [40], two methods were used to ostensibly distinguish striped morphologies. First, after plane-fitting the data, convolution with a 2D Mexican-hat wavelet (effectively a highly localised bandpass filter [59]) was used to highlight features of a specific chosen size [60], [61]. These were interpreted as ligand head groups. It is perhaps worth noting that the wavelet convolution used is described as a continuous wavelet transform. This is incorrect, as the frequency is not allowed to vary [61], [62]. Instead, a particular spatial frequency of the wavelet was chosen by the user. The highlighted features were located using watershed analysis, marked in the manuscript images, and shown to form clusters.

We make two key points regarding this analysis. First, using watershed analysis on structure highlighted with the type of convolution approach employed by the authors will locate features in almost any image if the settings are adjusted appropriately. More importantly, clustering of point-like features is expected for a random (Poisson) distribution [63], [64]. No attempts to analyse the spatial distribution of the features — via, for example, correlation functions or Minkowski functionals [65] — to assess the degree of randomness is made. As mentioned previously, careful quantitative analysis is essential as humans instinctively recognise patterns where no true spatial correlation exists [51], [52].

**Figure 9:**
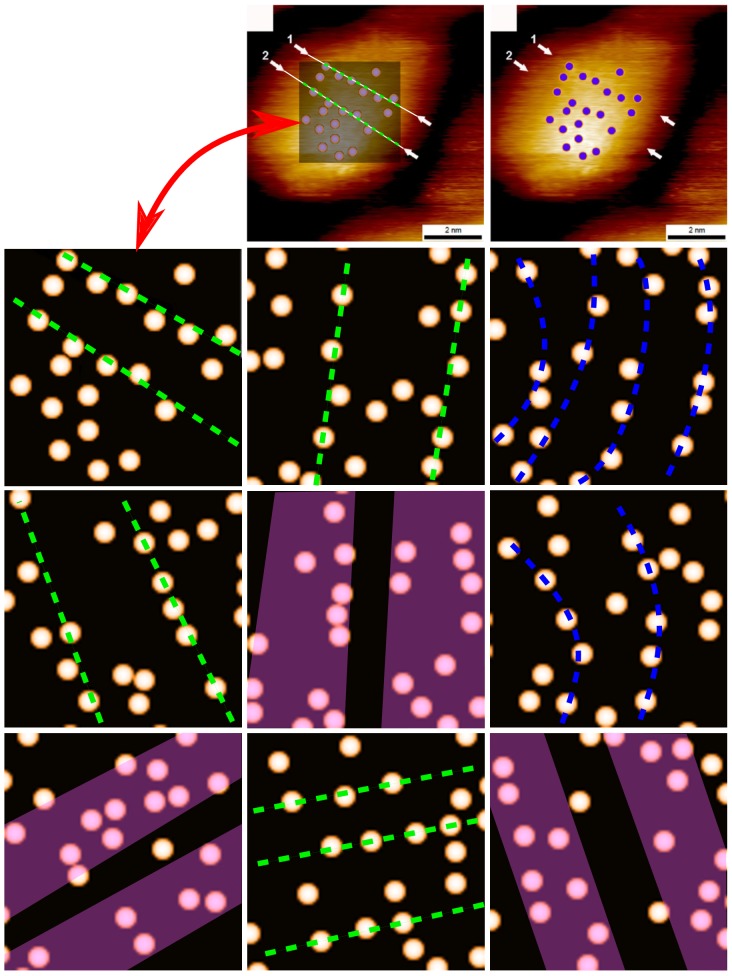
Digitised position of ligand head groups and stripes identified by Ong et al. [40] as compared to eight sets of randomly distributed ‘head groups’. The top panel is reproduced from Ong et al. (2013) High-resolution scanning tunneling microscopy characterization of mixed monolayer protected gold nanoparticles. ACS nano 7: 8529–39 (DOI: 10.1021/nn402414b) - by permission of the American Chemical Society. The top row shows the image in question from Ong et al. (upper right corner) along with a version of that image where we have superimposed a semi-transparent square and highlighted the ‘stripes’ identified by Ong et al. using green dashed lines. The original blue circles (right) are visible through the digitised head groups. The positions of the head-group features within that square, and the corresponding dashed lines highlighting the ‘stripes’, are then reproduced on a featureless background, as indicated by the red double-headed arrow. The other eight images in the figure for comparison show randomly distributed features. By either assigning straight lines (green), curved lines (blue), or stripe-like domains (purple) it is possible to guide the reader’s eye to clustering in random features.

To highlight this problem, in Figure 9 we compare the distribution of assigned head groups and striped domains from an image in Ong *et al.* [40] with randomly positioned particles. Note how the eye can very easily be tricked into finding patterns in particles which have zero spatial correlation. The code used to generate the randomly distributed particles and the distribution from Ong *et al.* is given in the public data archive [36].

The second method adopted by Ong *et al.* [40] to detect striped morphologies is to use a radially averaged 2D power spectral density (PSD) plot. The 2D PSD is the modulus squared of the 2D Fourier transform. A radially averaged PSD indicates the presence of oscillating features in *any* spatial direction. As this paper concentrates on images of single nanoparticles, where oscillations from stripes will have a particular orientation, radially averaging simply removes any directional information present in the 2D PSD. Figures 7 (d–f) correspond to radial PSDs of the same type of nanoparticle samples imaged in (a–c) of that figure respectively. (Note, however, that the PSDs are not taken from the images shown in (a–c)). The triangle and circle in Figure 7d) mark small peaks in the radial PSD when plotted on a logarithmic scale. These peaks are interpreted as corresponding to the spacing between head groups within stripes and the distance between stripes with distances of 0.59 and 0.83 nm, respectively. We note that even for a *square* grid of features, one would expect two peaks in a radial PSD corresponding to row spacings and diagonal spacings, with a ratio of 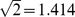. The ratio between spacings in Figure 7d) is 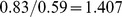 which agree with a square grid to 3 significant figures. We do not use this observation to imply that the features in the image are distributed on a square grid, but simply to point out that there are multiple possible interpretations of a radial PSD of point-like features.

The PSD analysis also suffers from other flaws. Ong *et al.*, use the line in Figure 7e) to define a wide peak corresponding to the distance between stripe-like domains. Remarkably, however, the two peaks present in Figure 7f), are not marked, despite being significantly stronger than those in Figure 7e). Those features are nonetheless mentioned in the text of the paper, where they are assigned to distances present in the randomly ordered ligand arrangement. This assignment begs the question as to why the peaks in Figure 7d) and e) could not arise from random ordering; why the full 2D data was not analysed to get directional information on these peaks; and why no mathematical analysis was applied to test for randomness in the located head group positions.

The radial PSD approach employed by Ong *et al.* [40], therefore, cannot be used to objectively determine whether stripes are present in the nanoparticle ligand shell. We now turn to a critique of the 1D PSD method used in a paper published shortly after that of Ong *et al.* where Biscarini *et al.* [41] apply a modified PSD method to quantitatively analyse both new and old STM images from Stellacci *et al.* In Biscarini *et al.*'s case, a 1D PSD is acquired by calculating the PSD for each scan line in the image and averaging down the slow scan direction. This method will capture stripes aligned with the scan direction while stripes of spatial frequency *f* misaligned by an angle 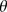 will appear at a frequency of 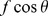. Thus, if 1D PSD analysis of this type is applied to an image with randomly aligned striped particles, one expects a broadened peak near the stripe spatial frequency (assuming that there is a sufficiently high number of particles in the image to produce a well-resolved peak).

When plotting the 1D PSD on a logarithmic scale, Biscarini *et al.* [41] observe an initial plateau and shoulder arising from the characteristic size of the nanoparticles, followed by a decay, then a second plateau and shoulder, followed by another decay. The second plateau and shoulder is, rather precipitously, taken as evidence for the striped morphology. Little time is spent by Biscarini *et al.* [41] to determine that this shape cannot arise from other image features. We show in the following that the plateau and shoulder do not arise from stripes, but from a random arrangement of features on the nanoparticle surfaces.

**Figure 10:**
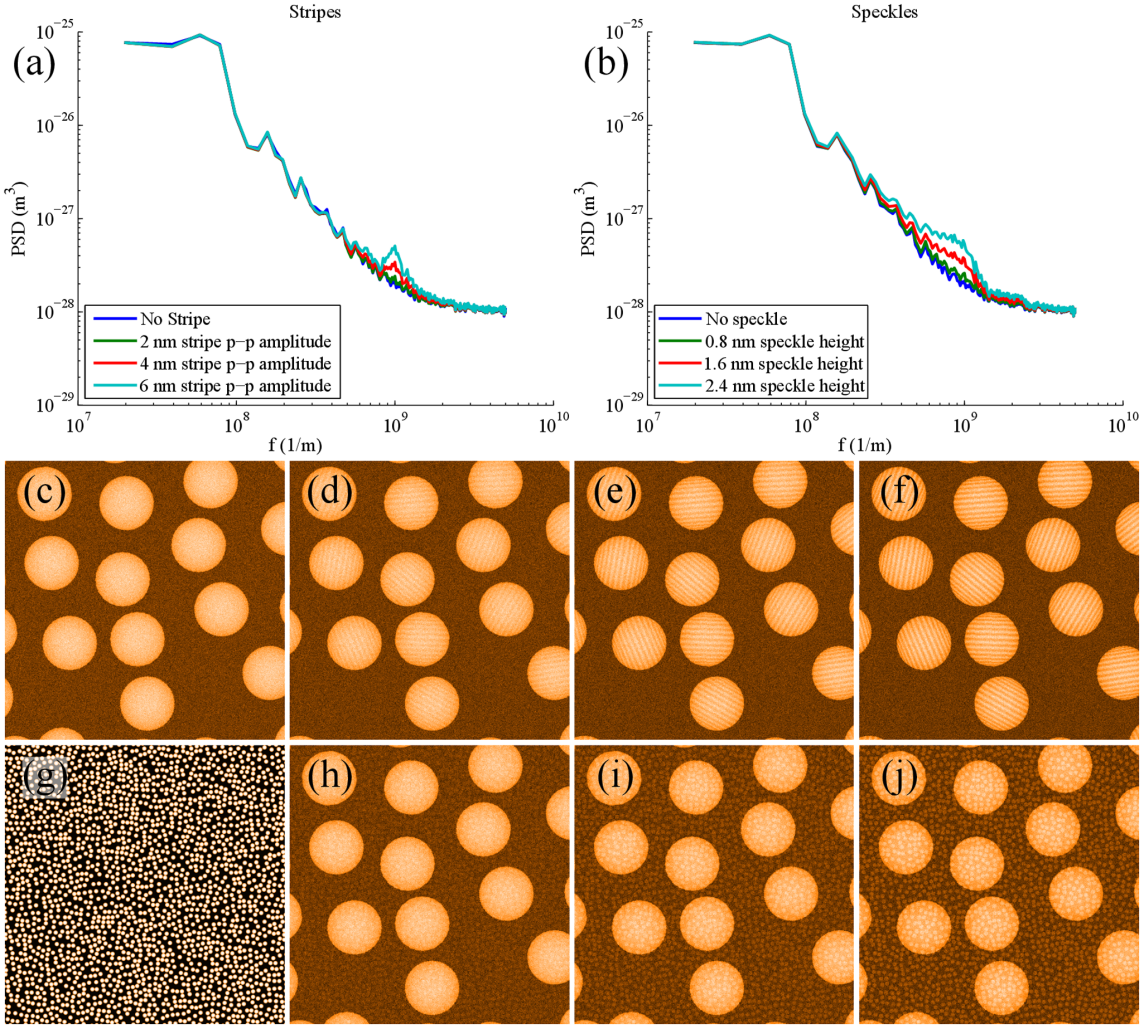
1D PSDs of simulated nanoparticle substrates. (a) 1D PSD for nanoparticles for simulated stripes of increasing amplitude (see simulated images shown in c–f); (b) Equivalent to (a) but in this case for simulated nanoparticles covered in randomly positioned speckles (ligand head groups (see images (h–j)). The speckled images simulating a random distribution of head-groups yield the plateau and shoulder observed by Biscarini et al. [41] which were inadvertently assumed to represent the signature of a striped morphology.; (c) Simulated flat surface with 10nm diameter spherical nanoparticles. (d–f) 1nm wide sinusoidal stripes are added to the surface of the nanoparticles (thus, they reduce in width at the edge) with peak-peak amplitudes of 2, 4 and 6 nm respectively; (g) Randomly distributed ‘speckled’ pattern of features 0.8 nm in diameter. (h–j) Images of simulated nanoparticles where the speckles in (g) have been added to (c) with heights of 0.8, 1.6 and 2.4 nm respectively. (c–f and h–j) have had identical white noise added for consistency and for a fair, unbiased comparison.

Figure 10c) shows a simulated nanoparticle substrate. If stripes are present on the particles (Figure 10d–f)), then the expected broad peak forms in the 1D PSD Figure 10a). We also note that the stripes are clearly visible to the eye before the 1D PSD peak becomes noticeable. If, however, randomly positioned speckles (Figure 10g)) are added to the substrate (Figure 10h–j)), the plateau and shoulder observed by Biscarini *et al.* in the experimental data are produced. Indeed, Biscarini *et al.* observe a very similar plateau and shoulder for homoligand nanoparticles, but they argue that because the shoulder appears at a different spatial frequency this distinguishes it from the structure in the PSD arising from the stripe-like morphology. This is an entirely unwarranted conclusion to draw and begs yet another question: why does the presence of the plateau-and-shoulder structure in the PSD at a different spatial frequency not lead to the natural conclusion that the PSD points to the presence of a similar (random) morphology, but at a different characteristic length scale? Biscarini *et al.* [41] do not address this exceptionally important point.

To further suggest that the STM images used for their analysis are artefact free, Biscarini *et al.* [41] fit the PSD to extract characteristic frequencies which should be unchanged under varying scan speed, similar to the analysis in Hu *et al.* [38], except using Fourier analysis. This analysis, however, is once again multiply flawed. First, if stripes are not clear to the eye (and because, as shown above, the 1D PSD cannot distinguish between stripes and other morphologies), even if the spatial frequencies are real, this does not represent evidence for a striped morphology. In addition, as for the data previously analysed in Hu *et al.* [38], and discussed above, due to the variation of multiple scan settings in addition to the scan speed the test is not rigorous.

An additional fundamental difficulty with the analysis presented in Biscarini *et al.* [41] is that the fitting procedure used to extract spatial frequencies from the PSD data is very far from robust. Furthermore, the description of the fitting process given by Biscarini *et al.* in their paper is misleading at times. We describe the difficulties with the fitting process in detail in the File S1. Here, we simply state the following: (i) there are seven free parameters in the fit. Multi-parameter fitting of this type is not at all well-suited to extracting reliable (and unique) spatial frequency values [66], [67], particularly when the fitting was carried out by Biscarini *et al.* [41] in the manner described in the File S1; (ii) sections of the PSD data were excluded from the fit by Biscarini *et al.*, without this exclusion being explicitly mentioned in the text of the paper [68]. Even if this were not the case, the initial choice of fitting parameters can substantially bias the output of the fitting algorithm; and (iii) we have repeated the fits in MATLAB and find that in all cases warnings for poor convergence were given.

As a final note on Biscarini *et al.*, the PSD analysis is repeatedly argued as the best method for measuring image features as it contains the “whole information content present in the image”, and as such, is unbiased. This is, however, not true, as much of the information content of an image is contained in the phase components, and by taking PSD from the Fourier transform all phase information is lost [69]. In addition, by *choosing* to average over a *particular* direction further information content in other directions is lost.

As the last paper to be considered in this section, we turn to Moglianetti *et al.* [29], where the role of scan rotation on liquid STM images of a new type of mixed-ligand-terminated nanoparticle (dodecanethiol: hexanethiol, 2∶1) was studied. The PSDs of the STM images are also compared to data collected using small angle neutron scattering (SANS). The nanoparticles reportedly showed no striped morphology when imaged in ambient conditions while the liquid STM images presented instead are argued to show “clear stripe-like domains” for the particles.

Although Figure 1 of Moglianetti *et al.* [29] shows arguably the most convincing images of nanoparticle sub-structure we have seen to date in the work of Stellacci and co-authors (the persistence of features in the trace and retrace images is particularly compelling), the paper, far from demonstrating the presence of “clear stripe-like domains”, provides no evidence for stripe formation. Once again, there is strong observer bias in the identification of “stripes”. We suggest that the reader compare the dashed lines used to highlight the presence of “stripes” in Figure 1(e) of Moglianetti *et al.* [29] with those shown in Figure 9 above, where the head-group features are randomly distributed. The majority of the scientific arguments in Moglianetti *et al.* [29] relate to the SANS data and to comparisons of the STM and SANS results. We therefore consider this work in more detail in the final section of this paper.

It would be remiss of us to leave the discussion of Moglianetti *et al.* [29] without highlighting a troublesome misconception regarding STM image acquisition. In their paper, Moglianetti *et al.* [29] claim that *“as one rotates the image, the tip approaches the sample from different directions, this in turn leads to a change in image resolution, due to variation in the convolution conditions and the asymmetry in tip shape”*. This statement betrays a fundamental misunderstanding of STM operation. Artifacts from improper feedback settings will indeed depend on the scan rotation, but convolution effects result from the orientation of the tip relative to the sample. This does not change when the image is rotated via a change in scan angle: *neither the sample nor the tip is physically rotated*. Instead, the direction of raster scanning is changed. Any convolution effects from the tip are, therefore, expected to rotate with the image, as noted above in the context of the discussion of Figure 8.

Before moving on to critique the evidence for stripes from techniques other than STM, we note that following submission of the original version of this manuscript, Mezour *et al.* [70] reported that they had also observed stripes in the ligand shell of nanoparticles, except that in their case *the particles were terminated by only one type of ligand.* This runs entirely counter to Stellacci *et al.*'s proposal that stripes form only via phase separation in the ligand shell. Moreover, there is clear evidence in Mezour et al.'s paper that the features they have interpreted as stripes similarly arise from a scanning artefact [71].

### Assessment of evidence for nanoparticle stripes from techniques other than STM

In this section we will briefly critique the evidence for striped nanoparticles from techniques other than STM. These span nuclear magnetic resonance (NMR) spectroscopy, transmission electron microscopy (TEM), SANS, and computational simulations. The data from Fourier transform infra-red spectroscopy (FTIR) studies have not been considered, despite Yu and Stellacci [42] citing FTIR data in their response to Cesbron *et al.* [37]. This is because the paper cited by Yu and Stellacci explicitly states that FTIR can be used only to screen for phase separation, but cannot distinguish between striped and non-striped morphologies.

#### Analysis of NMR spectroscopy data

Liu *et al.* [28] present a method using 1D and 2D NMR spectroscopy which they argue can identify the morphology of ligand shells for mixed-ligand nanoparticles (MLNs). The core data centres around three types of MLN with binary ligand mixtures. All three contain diphenyl thiol (DPT) as one ligand. The first nanoparticle type has a diameter of 4–5nm, with 3,7-dimethyloctanethiol (DMOT) and DPT ligand mixtures that are assumed to form random ordering. A second type has a diameter of 2.2–3 nm, with dodecanethiol (DDT) and DPT ligand mixtures that are assumed to form Janus nanoparticles. Finally, a type with a diameter of 4–5 nm, also with a mixture of DDT and DPT, is assumed to have a varying patchy morphology, which exhibits stripes at 1∶1 ratios.

For the development of the NMR methodology, the morphology of the MLNs is assumed to be already known from STM data. This is critical because, as we have discussed at length in the preceding sections, there is no evidence from the STM data to date that stripes form in the ligand shell. In addition, the STM images for the Janus nanoparticles clearly show pairs of separate nanoparticles which are close together, ringed as ovals and described as single nanoparticles. From the NMR data, no direct evidence for the existence of the stripes is presented. The question of the validity of the reasoning, however, is still relevant to the argument for or against the striped morphologies.

Unfortunately, we found the data yet again to be inconclusive, combined with some major flaws in some specific areas of analysis. For brevity we will only discuss the 1D spectra below, as this forms the core of the presented evidence. The 2D data are, however, discussed in detail in the File S1.

The primary information used from the 1D NMR spectra is the chemical shift of the aryl peak maximum. There are various pieces of information that are not considered or interpreted. In particular, the line caused by the alkyl ligands is not analysed, despite its changing position and pattern. In addition, linewidths and lineshapes are not analysed in any way (neither in the 1D nor in the 2D data), with the exception of a narrow aryl line. This line is interpreted under the assumption that the morphology is known to be striped, and via an indirect argument based on the reactivity of ligands in nanoparticles. Further details regarding this narrow aryl line are presented in the File S1.

The model used to explain changes in the chemical shift of DPT assumes a linear change from the bulk chemical shift to the chemical shift of DPT surrounded by the other ligand as the ratio of the second ligand to DPT is increased. This relation is referred to as “trivial” with no consideration that the chemical shift can depend strongly on possible changes in the local ordering of the phenyl rings relative to each other or on the mobility of the thiols, which will change with varying ligand ratios. This is because ring currents in the aromatic rings of DPT cause a highly orientation-dependent shift of the ^1^H NMR resonances as a function of the proton position with respect to the ring [72]. Further problems exist with this model [73], which are again addressed in detail the File S1.

Assuming the validity of this linear model, Liu *et al.* [28] continue to derive an equation for Janus particles, which they refer to as “rigorous”. However, at neither concentration limit does the equation tend to the expected values; this point is never addressed. The model is fitted to the experimental results, but close inspection shows that both initial and final point are below the fit, with central points above. This trend in the residuals strongly suggests that the model does not fully explain the data. Upon reading the full text it becomes clear that to generate this fit the second point was arbitrarily designated as an outlier to increase the 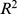 value. An 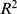 of 0.976 is used to suggest the model “provides excellent agreement” with no mention of the clear trend in the residuals [66]. In the File S1, we derive a revised model for Janus particles which provided a more accurate fit without any data exclusion. Our model still falls short of a rigorous model as it fails to converge if the mole fraction of the ligands being detected falls below the value necessary to maintain two bulk regions. Then all of the corresponding ligand molecules are located in the interface region, for which case the model is not designed to make any predictions. This point is not raised to dispute the evidence for Janus particles (although the STM data are not compelling), but simply to demonstrate further evidence of inadequate data analysis.

The key conclusion of our re-analysis of the NMR data, however, is that the evidence presented for striped morphologies is exceptionally weak. Liu *et al.* [28] suggest that for patchy nanoparticles with stripes around 1∶1 ratios, the chemical shift should vary as a sigmoidal function for increasing concentrations of DPT. This reasoning is not explained in their paper. As the change in chemical shift is dependent on the complex and unknown evolution of the patches a sigmoidal function cannot be assumed *a priori*. Similarly, no justification that other morphologies could not produce a sigmoidal function is given. In addition, the results do not unambiguously show a sigmoidal pattern. Instead, up to a DPT concentration of about 60% the chemical shift changes very little, followed by an almost linear reduction towards the bulk value. These data could also be equally well explained by the formation of small circular patches of DPT among DDT. As the concentration of DPT is increased more patches of similar size are generated, until a critical point is reached where the patches coalesce (see File S1 for more details). Liu *et al.* [28] instead use the large uncertainties in the measurement to claim that the straight lines are not statistically significant and that the true dependence may well be sigmoidal, and hence the data would be in “excellent agreement” with the striped model. This argument can be used to claim that the data do not preclude the possibility of a striped morphology, yet cannot be used as direct evidence in favour.

#### Analysis of TEM data

**Figure 11:**
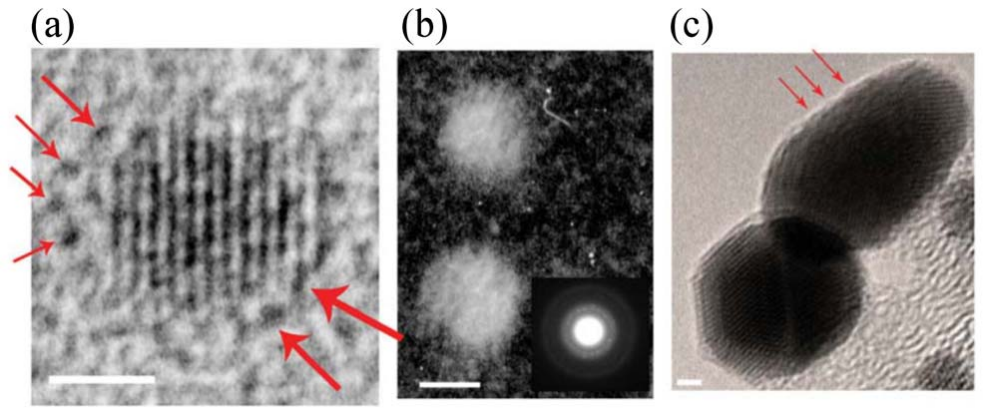
TEM data of OT:MPA-coated nanoparticles from Figure S2 of Jackson et al. [23] - Jackson et al. (2004) Spontaneous assembly of subnanometre-ordered domains in the ligand shell of monolayer-protected nanoparticles. Nature materials 3: 330–6. (DOI:10.1038/nmat1116) Reproduced by permission of Nature Publishing Group. (a) Red arrows indicate dark features surrounding the nanoparticle which have been interpreted as MPA head groups. Such features commonly arise from TEM defocus, and even if real are not arranged in striped domains. (b) Dark-field TEM image with inset power spectrum. (c) Further TEM image of mixed-ligand nanoparticles with red arrows supposedly indicating sinusoidal features. Neither (b) nor (c) show any evidence for an ordered striped morphology.

The TEM data cited as evidence of ligand stripes comprises just three images of OT:MPA MLNs in the supplementary information of Jackson *et al.* 2004 [23], Figure S2, reproduced in here in Figure 11. Taking (b–c) first, a dark-field and a bright-field image respectively, each shows two nanoparticles and neither show any evidence of stripes. The red arrows in (c) are reported to show sinusoidal features at the edge of the particle. For (a), single dark features around the particle are indicated with arrows, and were assumed by Jackson *et al.* [23] to be single MPA head groups. Our objection to this evidence is two-fold. First these features are of similar size to features in amorphous background, yet darker. A ring of such features is often seen in TEM images of bare nanoparticles [74]–[77], and can be enhanced or removed by varying the defocus [78], [79]. More fundamentally, even if these 6 to 7 features did represent MPA head-groups their ordering is in two groups, which may suggest phase separation, but does not show a striped morphology.

#### Analysis of SANS data

SANS data were presented as evidence for striped morphology on MLNs coated with dodecanethiol (C12) and hexanethiol (C6) in Moglianetti *et al.* [29]. The STM evidence from this paper has been considered above. In short, the SANS data similarly do not provide compelling evidence for the highly-ordered striped morphology claimed by Stellacci and co-workers. First, SANS results supporting the presence of these striped features require data acquisition at angles beyond the range measured by Moglianetti *et al.* Second, the highly-ordered stripe pattern which Stellacci et al. have repeatedly put forward on the basis of (inadequate) STM measurements, has not been retrieved from the SANS fits. Instead, the most likely morphology at best comprises a ‘fishnet-like’ segregation of the molecules. We will elaborate on each of these points in the following paragraphs.

Although it is clear that the SANS measurements have been performed with due care, the data quality for scattering beyond 2.7 nm^−1^ leaves a lot to be desired and precludes the accurate determination of features smaller than ∼2 nm. This is particularly unfortunate as the reported regular 1.2 nm spacing of the ‘stripes’ proposed to be on the stripy nanoparticles should have generated a striking feature at high Q in the collected SANS patterns.

A well-executed SANS experiment (consisting of an appropriate measurement, correction and analysis sequence) can be used to retrieve at most the element density distribution uniquely for a given sample. Any further conclusions (such as 3D structure or size distribution retrieval), are therefore only obtained with the provision of constraints provided through information from supplementary techniques. In other words, of the three aspects often sought — i.e., shape, polydispersity, and packing — two must be known (or assumed) in order to extract information on the third from scattering experiments [80]. In the work of Moglianetti *et al.* [29], the packing (dilute) and the polydispersity (monodisperse) are fixed so that the shape may be retrieved.

Moglianetti *et al.* impose further constraints through adjustment of the parameters of a dummy atom model (DAM) used for fitting of the SANS patterns. This model is part of the suite of software tools provided by Svergun *et al.*, which has been applied successfully to retrieve 3D structures for monodisperse biological structures, for example by Blanchet and Svergun [81] and by Vestergaard *et al.* [82]. In this particular implementation, a regular, immutable lattice of dummy ‘atoms’ (DAs), each 0.2 nm in size, is allowed to vary in choice of moiety (i.e. any DA can be gold, C6, C12, or solvent). DAs within a distance of 2 nm from the centre are fixed to the gold phase; between 2 nm and 3 nm from the centre, the DAs are either gold, C6 or C12; and from 3–3.8 nm they can only assume the C6 or C12 phase [29]. Through a simulated annealing search method that swaps the moieties of individual DAs, a close agreement with experimental data may be obtained.

Three configurations are considered for this particular application of the fitting routine: one where only cup-shaped C6/C12 phase segregation is allowed (forming Janus particles), one with a perfectly random distribution of moieties, and one where interconnected compact phases are imposed. Of these three models, the latter appears to describe the experimental SANS data best, though it must be stressed that other models may fit as well as, or better than, the models considered here. Agreement with a particular model can by no means be considered to provide a unique solution in small-angle scattering, as demonstrated by Rosalie and Pauw [83]. Similar conclusions are reached in the File S1 with regard to the NMR spectroscopy data of Liu *et al.* [28].

The small polydispersity of the gold nanoparticle cores (with a mean of 4.8 nm and a standard deviation of 0.4 nm) is not included in the fitting procedure. Consideration of polydispersity in the model fitting would further smear out the features observed in the models, and has the potential of reducing the agreement between the interconnected compact phase model and the SANS data. Its consideration would likely alter the retrieved shape significantly, as indicated by Filippov *et al.* [84].

Notwithstanding these issues, the methods employed do not result in a clear ‘striped’ phase segregation at all. Given the previously reported successes of the 3D DAM methods to retrieve complex biological structures, it would be expected that equally good agreement is attainable between the suggested shape and the SANS results. Indeed, it is remarkable that a simulated scattering pattern of the proposed highly-ordered striped morphology is not included for comparison. If, then, the fishnet morphology is representative for the moiety distribution across the nanoparticle surface, it stands in stark contrast to (and could in fact be considered strong evidence against) the very regularly spaced, well-aligned striped features claimed by Stellacci *et al.* throughout their work [23].

The last section of the paper by Moglianetti *et al.* discusses the comparison of PSDs. To take several of the 3D DAM structures, place them side-by-side, and then Fourier transform them (ostensibly allowing better comparison between the SPM images and the SANS results), is a rather uncommon method. Effectively, this methodology takes data from reciprocal space (the SANS data, collected from billions of nanoparticles), resolves a 3D real-space structure using the DAM method, places several retrieved structures side by side, only to put this information back in reciprocal space again (PSD). There are so many pitfalls imaginable in this methodology, that it first needs to be demonstrated to work at all before any judgement on its usefulness can be made here. Furthermore, the characteristic length extracted for the SANS-derived and STM PSDs differs by 50%.

#### Comment on computer simulations

To discuss the evidence for striped morphologies from computer models one must understand the role of simulations as an aid to understanding experimental data and making predictions from theories [85]. Simulations all come with their own advantages and difficulties, and, depending on what information is desired, different methods are applicable. For predicting structures, methods closest to *ab initio*, such as DFT, are usually preferable. Such simulations are computationally expensive and thus are only performed on relatively small numbers of atoms. Statistical methods such as Monte Carlo simulations [86] [87], or semi-classical approaches such as molecular dynamics [88], are less expensive and thus larger systems can be studied, but at the cost of decreased accuracy.

The bonding of thiols to Au surfaces is still not completely solved [89], but our understanding has improved vastly since the original simulations of striped morphologies on Au nanoparticles [30]. The prevailing view was that the thiols bond through the sulphur to the Au surface at a specific site [90]. More recent studies, however, indicate that thiols bond as Au-adatom-dithiolate structures (R-S-Au-S-R), with strong supporting evidence from DFT simulations [89], STM on Au surfaces [91], and on Au nanoparticles via x-ray diffraction [92]. Further DFT and XPS studies have shown variations in binding energy arising from interactions between dissimilar thiols [93].

The simulations presented as evidence for the striped morphology use a mesoscale simulation called dissipative particle dynamics [94]. Here intramolecular interactions are modelled as harmonic springs [30]. Intermolecular interactions are treated as harmonic potentials with the model parameters chosen to have a higher repulsion between atoms on unlike molecules. Ligand-Au bonding is not modelled. Instead, constrained dynamics are used to confine the head group to a sphere. This form of large scale simulation, due to the simplicity of the interaction and the unknown accuracy of the chosen parameters, cannot be used to reliably predict the complex structures on coated nanoparticles. It is instead used to search for experimentally known structures. Once these structures, and their evolution under changing conditions, can be matched to the outputs of the simulation it is possible to extract theoretical understanding of the observed structures. Further simulations were also performed using molecular dynamics with a similar constrained geometry, and selected potentials instead of repulsion parameters [30].

This approach to modelling not only simplifies bonding and molecular interactions, it also simplifies the structure of the nanoparticle itself. Nanoparticles capped in thiols are known to be more spherical than bare nanoparticles due to thiol interaction [95], but faceting is still present on the nanoparticles [92]. In addition, it is known that thiols modify gold surfaces [89] and nanoparticles [88] during the formation of self-assembled monolayers. Furthermore, the simulations only deal with the rearranging of randomly ordered thiols, not the posibility of structures arising from selective adsorption or ligand exchange [96], [97].

These criticisms of the simulation are not meant to suggest that the simulation was poorly performed or is unjustified due to its simplicity. If a simple simulation can accurately describe and provide insight into experimentally observed behaviour then it is a valid simulation. However, *if the experimental evidence for the structure is called into question it is tautological to use a simplistic simulation designed to understand this structure as evidence that the structure itself does exist.*

## Conclusions

We have critiqued and re-analysed the extensive series of papers from Stellacci *et al.*, which argue that stripes form in the ligand shell of appropriately functionalised nanoparticles. The experimental evidence required to justify the claim of striped morphologies is lacking. Moreover, the majority of the published data suffers from rudimentary flaws due to instrumental artefacts, inappropriate data acquisition and analysis, and/or observer bias. The first paper claiming to resolve ligand stripes, Jackson *et al.* 2004 [23], shows features which arise from feedback instabilities and which can be reproduced on bare nanoparticles. Jackson *et al.*'s results were supplemented with papers which attempted to differentiate between artefacts and true nanoparticle topography on the basis of the variation of scan parameters. The methods used in these studies are far from rigorous, as multiple conditions changed between images. Moreover, the division of similar image features into artefacts and ‘true’ striped features was performed by a human operator with no rigorous selection criteria. Recent STM data, collected in collaboration with other SPM groups, despite being taken at significantly higher resolution shows a significant decrease in sub-nanoparticle contrast. The reduction in contrast is so strong that the stripes cannot easily be recognised in real space. To investigate the stripes Fourier space analysis has therefore been applied. We show, however, that the Fourier space techniques which have been employed are unable to reliably discriminate between stripes and other morphologies. Finally, the quantitative methods, which have previously been developed for extracting spatial frequencies from the resulting Fourier space data, are fundamentally flawed as they rely on a multi-parameter fit, which is highly sensitive to the initial, user-defined, fitting parameters. On the combined basis of our analysis of the flaws in the scanning probe studies and our criticisms of the evidence from other complementary techniques, we conclude that no reliable evidence has been presented to date for the presence of ligand stripes on mixed-ligand nanoparticles.

## Supporting Information

File S1 **This supplementary information file details how to use the code (available from Reference [36]) to generate the figures presented in the main paper.** It also contains extra information on the flaws in both the NMR spectroscopy anaylsis of Liu *et al.* [28] and Biscarini et al.'s [41] fitting of 1D power spectra curves which was not included in the main text for brevity.
(PDF)
