# Supplementary material for: Critical Assessment of the Evidence for Striped Nanoparticles
Source: PLoS One. 2014 Nov 17;9(11):e108482. doi: 10.1371/journal.pone.0108482 (PMC4234314; doi:10.1371/journal.pone.0108482)
Supplement: File S1 — This supplementary information file details how to use the code (available from Reference [36]) to generate the figures presented in the main paper. It also contains extra information on the flaws in both the NMR spectroscopy anaylsis of Liu et al. [28] and Biscarini et al.'s [41] fitting of 1D power spectra curves which was not included in the main text for brevity. (PDF) [file pone.0108482.s001.pdf]

Critical assessment of the evidence for striped nanoparticles: Supplementary Information

Julian Stirling[1,*], Ioannis Lekkas[1], Adam Sweetman[1], Predrag Djuranovic[2], Quanmin Guo[3], Brian Pauw[4], Josef Granwehr[5], Raphaël Lévy[6], Philip Moriarty[1]

1 School of Physics and Astronomy, The University of Nottingham, Nottingham, United Kingdom

2 Department of Materials Science and Engineering, Massachusetts Institute of Technology, Cambridge, MA, USA

3 School of Physics and Astronomy, University of Birmingham, Birmingham, United Kingdom

4 International Center for Young Scientists (ICYS), National Institute for Materials Science (NIMS), Tsukuba, Ibaraki, Japan

5 Sir Peter Mansfield Magnetic Resonance Centre, School of Physics and Astronomy, The University of Nottingham, Nottingham, United Kingdom

6 Institute of Integrative Biology, University of Liverpool, Liverpool, United Kingdom

∗ E-mail: Julian.Stirling@nottingham.ac.uk

Contents

1 Overview of supplementary information 2

2 Analysis of Figure 1 from Jackson *et al.* 2004 (Figure 1) 2

3 Feedback loop instabilities (Figure 2) 2

4 Parameter dependent imaging (Figure 3) 3

5 Reanalysis of Jackson *et al.* 2006 (Figure 4) 4

6 Pixelated data (Figure 5 and 6) 5

7 Figure 7 6

8 Persistence of tip-induced features (Figure 8) 6

9 Clustering of randomly positioned particles (Figure 9) 7

10 Features in 1D PSDs (Figure 10) 7

11 Further detail on fitting in Biscarini *et al.* 7

12 NMR spectroscopy 11

12.1 Further details on 1D spectroscopy of broad aryl peak . . . 11

12.1.1 Further details for Janus nanoparticles . . . 11

12.1.2 Further details for patchy/striped nanoparticles . . . 13

12.2 Discussion of narrow aryl peak . . . 14

12.3 Discussion of 2D NOESY data . . . 15

12.4 Section summary . . . 15

1 Overview of supplementary information

This supplementary information file details how to use the code (available from Reference [1])[1] to generate the figures presented in the main paper. It also contains extra information on the flaws in both the NMR spectroscopy anaylsis of Liu *et al.* [2] and Biscarini et al.'s [3] fitting of 1D power spectra curves which was not included in the main text for brevity.

2 Analysis of Figure 1 from Jackson *et al.* 2004 (Figure 1)

Figure 1 was created using raw data from Jackson *et al.* 2004 [4], part of the public archive released by Stellacci and co-workers in May 2013. This file is located at: `Public Data/Nature Materials 2004/Fig 1 original rippled files (ajs1)/raw files/npmono_gold_aj.006`

The full code to generate Figures 1(a,b,d,e) is provided in the file `NatureMat2004/Cut_n_Filter.m`. Figure 1(c) is from Jackson *et al.* 2004.

3 Feedback loop instabilities (Figure 2)

A real STM controller records the tunnel current at a specific sample rate rather than continuously, and all feedback calculations are discrete rather than analytical. As such, we argue it is most appropriate to model the STM system numerically.

For this we have decided, for speed and simplicity, to simply feed back on the height error, rather than convert a height into an exponentially decaying current and then take the logarithm. For this simulation the heights and gains are arbitrary. An algorithmic explanation of the feedback simulation is provided as pseudocode in Algorithm 1. The full simulation can be run from the file `Feedback/RunSimulation.m`, which requires the SPIW MATLAB toolbox [5], and the provided functions:

- `Feedback/GenNanoparticle.m`
- `Feedback/placeinpos.m`
- `Feedback/SimulateSPMFeedback.m`

Navigate to the Feedback directory before running. Run `doc functionname` for the help file of a particular function.

Final images are bicubically interpolated to a higher number of pixels to match the presentation of Jackson *et al.* 2004 [4]. In the folder we provide example outputs for those who do not have MATLAB. An example output is shown in Figure S1. For these outputs the top image shows the surface, the second row show the images before interpolation, and the bottom row shows the final interpolated images. Feedback settings are provided in the output image title.

An advantage of this numerical simulation is that it allows us to more accurately replicate the conditions of a real STM. Arbitrary topographies can be used, allowing us to build nanoparticle surfaces to scan, and add normally distributed noise to each measurement to simulate electrical noise. There are options to change the proportional and integral gains, the scan speed, and the set-point height. Additional options include changing the sample rate of the STM controller and changing the amplitude of the normally distributed noise. Finally one can turn on wind-up protection mode where the integral term in the feedback controller is reset after each pixel. (This mode tends to remove stripes or, in the case of very high integral gain, to 'lock' stripe widths to 1 pixel.)

[1] All code is released under FreeBSD licence, licence is included in root directory.

Algorithm 1 Pseudocode for STM simulation algorithm. Braces indicate comments.

Require: The topography *topog* (an $N \times N$ array), time per line $Tline$, Sample Rate SR, Set-Point $SetPoint$, Proportional gain $Pgain$ and Integral gain $Igain$, Wind-up protection (boolean) $Protect$.
Ensure: The simulated STM outputs *scan* and *scanR*.

```
spp = ROUND(Tline*SR/N) {Calculate the number of PID iterations taken per pixel}
height = topog(1,1) + SetPoint
I=0 {Initialise integral term}

{Using loops to simulate raster scan}
for m = 1 to N do
  for n = 1 to 2*N do
    if Protect then
      I = 0 {Reset integral term if wind-up protection is on.}
    end if
    {Calculate if on trace or retrace}
    if n > N then
      n2 = 2*N + 1 − n; {If on retrace, new fast scan position is calculated from n}
    else
      n2 = n; {If on trace fast scan position is simply n}
    end if
    for i = 1 to spp do
      {Calculating error and adding normally distributed noise}
      err = topog(m, n2) + SetPoint − height+ RANDN
      I = I + err
      {Using feedback to adjust height.}
      height = height + Pgain*err + Igain*(I/SR)
    end for
    {Scan data is the last height, must be written to either scan or scanR depending on if tip motion
    is trace or retrace}
    if n > N then
      scanR(m, n2) = height
    else
      scan(m, n2) = height
    end if
  end for
end for

return scan, scanR
```

4 Parameter dependent imaging (Figure 3)

Figure 3 shows results for data collected on an Omicron LT at 77K in UHV on bare Ag nanoparticles as described in the main text. The raw data for all images in this Figure is provided in the folder `NewData/RawData`. The full experimental data collected is not provided due to its large size (over 5 GB) but is available on request.

The first two rows show consecutive retrace-down images with incremented gain between each image. This part of the figure is generated by the file `NewData/IncGain.m`. All images have been bi-cubically interpolated to four times the original pixel density in both dimensions. In addition, the particles have been aligned using cross-correlation to correct for drift between images. To compare the trace and retrace

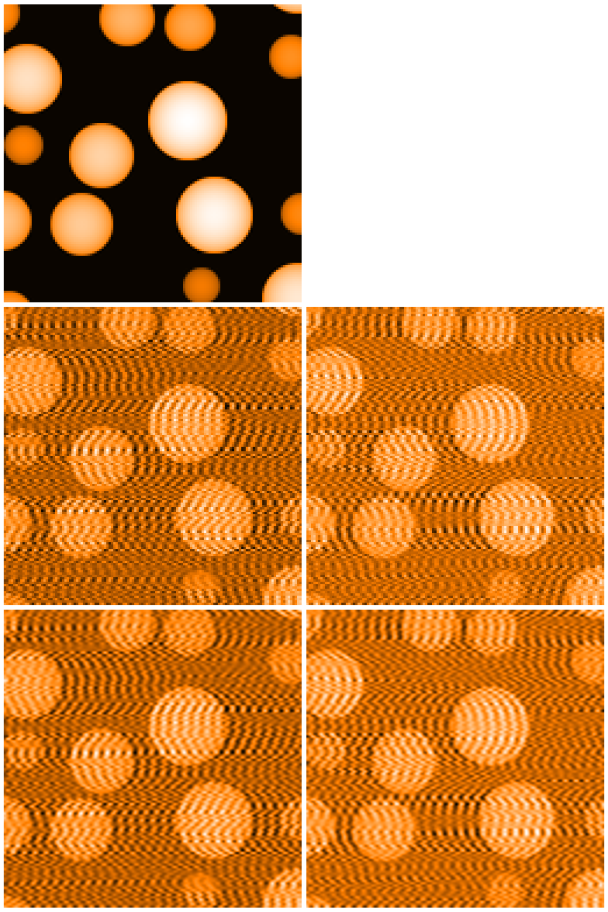

Figure S1. Example output from the program `Feedback/RunSimulation.m`. Top row shows the surface. Middle row trace (left) and retrace (right) before interpolation. Bottom row trace (left) and retrace (right) after interpolation.

of a similar data set, see the file: `NewData/IncGain2.m` and the image output: `NewData/IncGain2.png`.

5 Reanalysis of Jackson *et al.* 2006 (Figure 4)

Images from Jacskon 2006 were provided in the archived data provided by Stellacci and co-workers and can be found in the directory: `Public Data/Fig.3 Jacs 2006`. The analysis which produced Figure 4 is provided in three scripts which should be run successively:

1. `NewData/LineByLineAnalysis.m`
2. `NewData/make_compare_graph1.m`
3. `NewData/make_compare_graph2.m`

The code is well commented, but we provide an overview here for those who are not particularly familiar with MATLAB.

Before MATLAB processing, we have digitised the graphs for Figure 3 of Jackson *et al.* 2006 [6] using Engauge Digitizer. The `.txt` export, the `.png` image, and the `.dig` save file for each of the three sub figures are provided in the directory `NewData`.

First, we open the current image for each provided file in turn, as the current image should be dominated by any feedback effects. We then perform a Fourier transform on each fast-scan line and take its modulus to get a power spectrum for each line. We then take the median of the power spectrum for each frequency to provide an averaged power spectrum. The reason for taking a median is that as the feedback is unstable it is possible to have spurious line with a particularly high or low power at a particular frequency. The median allows us to remove the effect of spurious outliers. Figure S2 shows a plot of this resulting averaged power spectrum plotted on a linear scale. The broad peak from the feedback instabilities is clear. Just below the legend horizontal lines are plotted showing the frequencies and errorbars for the "noise" from Jackson *et al.* 2006 [6].

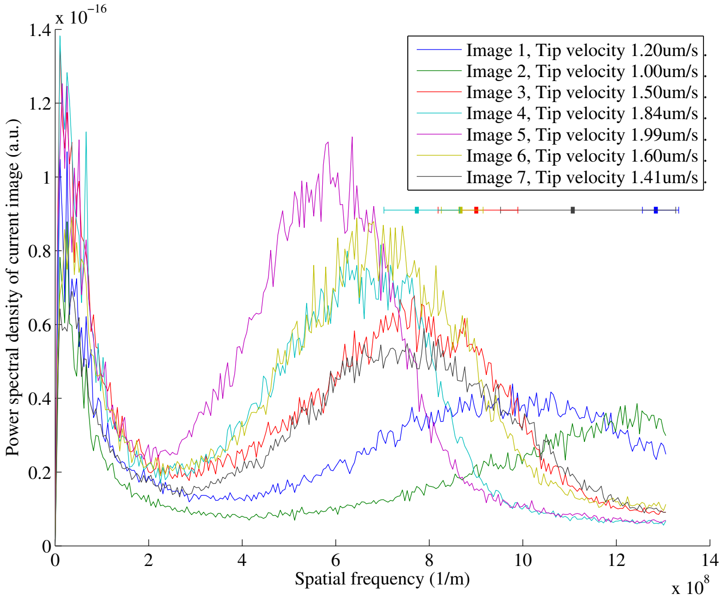

Figure S2. Median power spectra for the archived data provided from Jackson *et al.* 2006 [6]. Horizontal lines below legend represent range of values for "noise" from Jackson *et al.* 2006.

To extract the full width at half maximum we are aware that the frequencies for some images are greater than the Nyquist frequency. We therefore calculate instead the half width at half maximum from the low frequency side of the curve. This is then used as the upper and lower error bar. To do this we first truncate the data to remove the low frequency components, then apply a 31-point boxcar average (31 points were chosen to produce a smooth curve without significantly affecting the shape of the overall curve) to the data and then locate the global maximum. From the global maximum we search for the first point lower than half the maximum value on the low frequency side. The smoothed data and located maxima and half maxima are shown in Figure S3

6 Pixelated data (Figure 5 and 6)

The data from Yu and Stellacci were not provided in the public archive of data but were sent (by Prof. Yu) to one of the authors (PJM) via private e-mail communication. Unfortunately, however, the parameter file for the experiments was not included in the data sent by Yu to PJM. The parameter file for Omicron

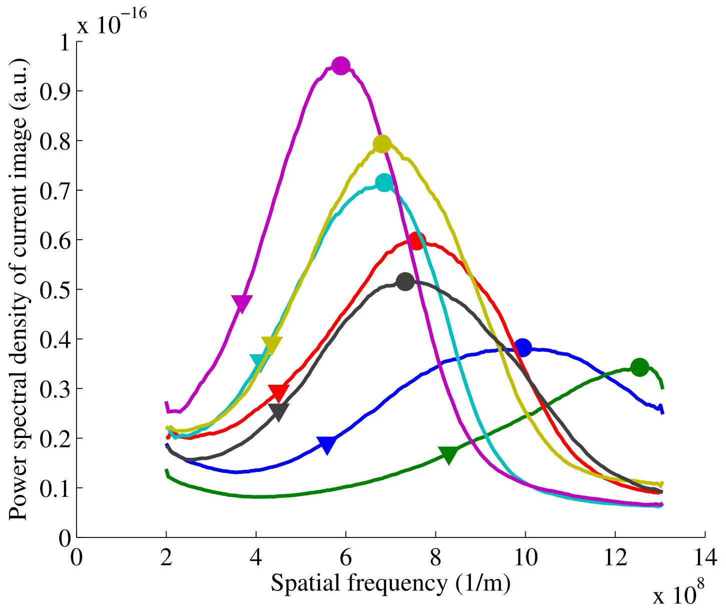

Figure S3. Truncated median power spectra after a 31-point boxcar average. Located maxima and half maxima are shown with circles and triangles respectively. Colours are consistent with Figure S2.

experiments contains all meta-data for each image, including the width, height, scaling for the z axis, tip speed, and all other imaging parameters. These files could not be opened with SPIW due to the lack of this file. A different software package, Gwyddion, can open them (assuming a square image) and produces images with an arbitrary z scale and unknown dimensions. ASCII exports of this data, from Gwyddion, was used for processing this data in MATLAB. The images are known to be 80 nm in width from provided `.tiff` files. All raw and exported data is provided in the directory `Small`.

The method used to process Figures 5 and 6 are detailed in the main text. The code used is provided (in a commented form) in the files: `Small/AddIms.m` and `Small/MakeFigure.m` respectively. This relies on a provided function:
`Small/compare_trace_small_2012.m`.

In this directory there is also an interactive tool (`Small/InvestigateGUI.m`) to simultaneously view the same section of trace and retrace image for any of the scans provided by Yu and Stellacci. This tool shows both images before and after interpolation. The zoom size, position, and contrast can be modified using a GUI interface.

7 Figure 7

Figure 7 is entirely produced from figures in Ong *et al.* [7] with no further processing.

8 Persistence of tip-induced features (Figure 8)

Figure 8 was produced from the dataset described above for Figure 3 (Section 4). No image processing except first order plane flattening is applied to these images. The code used to export the images is provided in the file `NewData/BecomeJanus.m`.

9 Clustering of randomly positioned particles (Figure 9)

The top left digitised image was produced in GIMP by overlaying the features used in other images onto Figure 2(b) of Ong *et al.* [7]. A higher resolution copy of this overlay is provided in Figure S4 with the partial transparency of our figure showing the blue circles from the original figure. The other 8 panels were produced by randomly placing features (rejecting any that overlapped). The code used to generate these is provided in `ACSNano_n_Langmuir2013/Random_speckle.m`

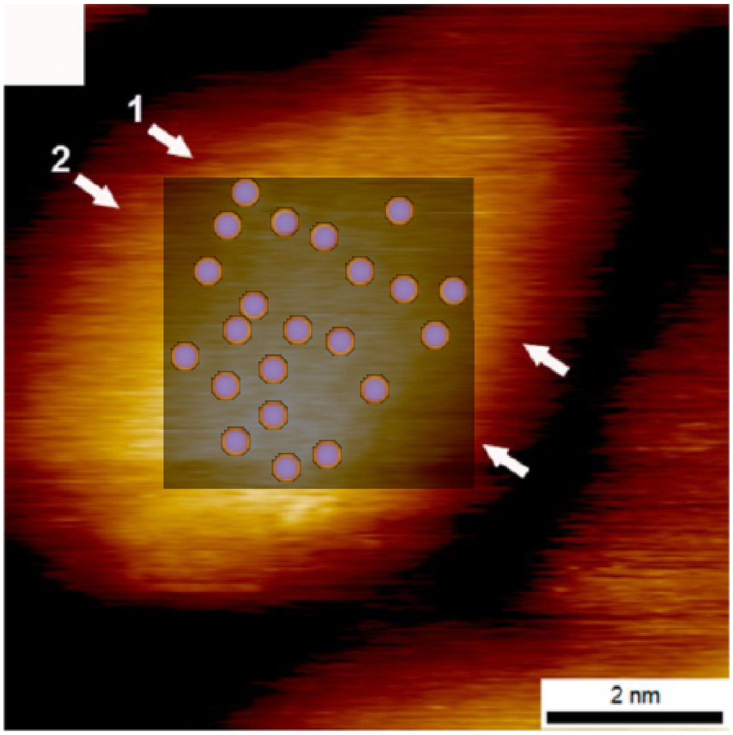

Figure S4. The top left panel of Figure 7 from the main text, semi-transparent and overlaid on Figure 2(b) of Ong *et al.* [7]

10 Features in 1D PSDs (Figure 10)

The code used to generate Figure 10 is provided in the file:
`ACSNano_n_Langmuir2013/PSD_stripy.m`. This file uses the function `Speckle.m` (also used for Figure 9) in the same directory and also functions in the `Feedback` directory used to create the surface for the feedback simulation.

11 Further detail on fitting in Biscarini *et al.*

The approach for extracting spatial frequencies in Biscarini *et al.* [3] involves a seven parameter fit to a function which assumes exactly two plateaus and ther associated decays (refereed to as shoulders) added to 1/f noise. Biscarini *et al.* argue that the first shoulder arises from the spatial frequency of the

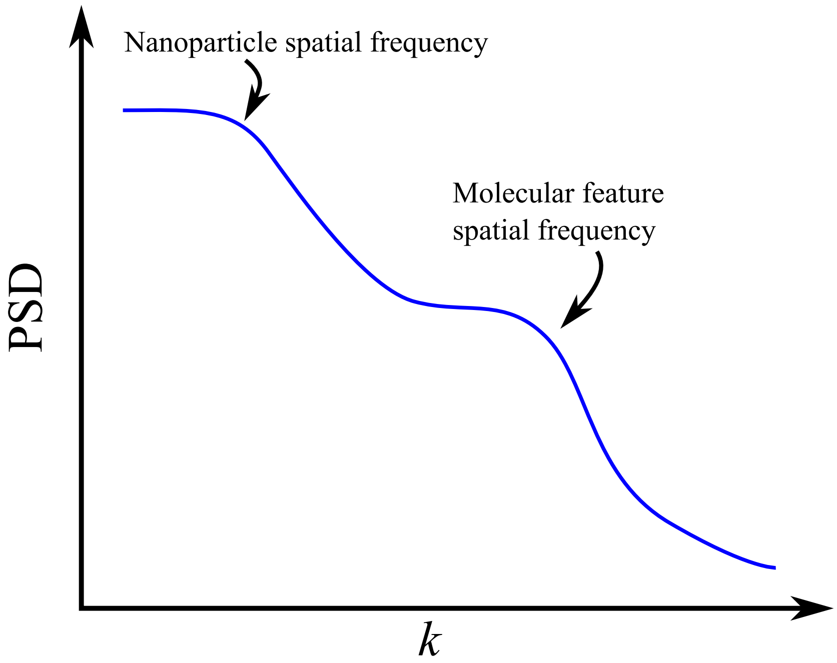

Figure S5. Cartoon illustrating the proposed functional form and its interpreted meaning. A seven free parameter function with two shoulders followed by a 1/f decay is used to model the PSD in Biscarini *et al.* The first shoulder is assumed to give the characteristic spatial frequency of the nanoparticles. The second shoulder is assumed to give the characteristic spatial frequency of molecular features on the nanoparticles. We emphasise that the figure is a cartoon and not a calculated function.

nanoparticles, and the second shoulder arises from the molecular features, after the second shoulder there is a decay due to noise (Figure S5).

It is clear from Figure 10 of the main text that the two shoulder PSD does not indicate stripes. It simply indicates features at a certain spatial frequency. We accept that if the noise level is higher, striped features may show this two shouldered structure, but it is important to remember that this method is not selective, and **other features also produce two shoulders**. In fact, Biscarini *et al.* take PSDs of homoligand (non-striped) nanoparticles and see this structure (Figure S6(a)).

Biscarini *et al.* instead argue that they can use the consistency of the position of the second shoulder to show that images from multiple labs, and taken over multiple years show the same features, ignoring the possibility that these PSD features may arise from features that are not stripes. First they present data taken in different labs in 2012/2013 (Figure S6(b)). We cannot see how the blue curve (taken from data acquired in the group of Christoph Renner, Geneva) and the green curve (from the results of de Feyter's group at Leuven) show any agreement to the others

Figure S6(c) shows data presented from Jackson *et al.* 2004 [4]. The vertical lines supposedly show the position of the second shoulder extracted from each curve. The red curve is discarded as an outlier and the other 3 apparently show agreement. Also, it is not immediately clear why the spatial frequency of the black curve is located so far from the clear shoulder at a much higher spatial frequency. To investigate this anomaly we re-applied the fitting method described in Biscarini *et al.*, as non-linear fitting using such a high number of parameters, and especially those containing power laws [8, 9], are very sensitive to initial conditions and can give results which vary strongly depending on the choice of starting values.

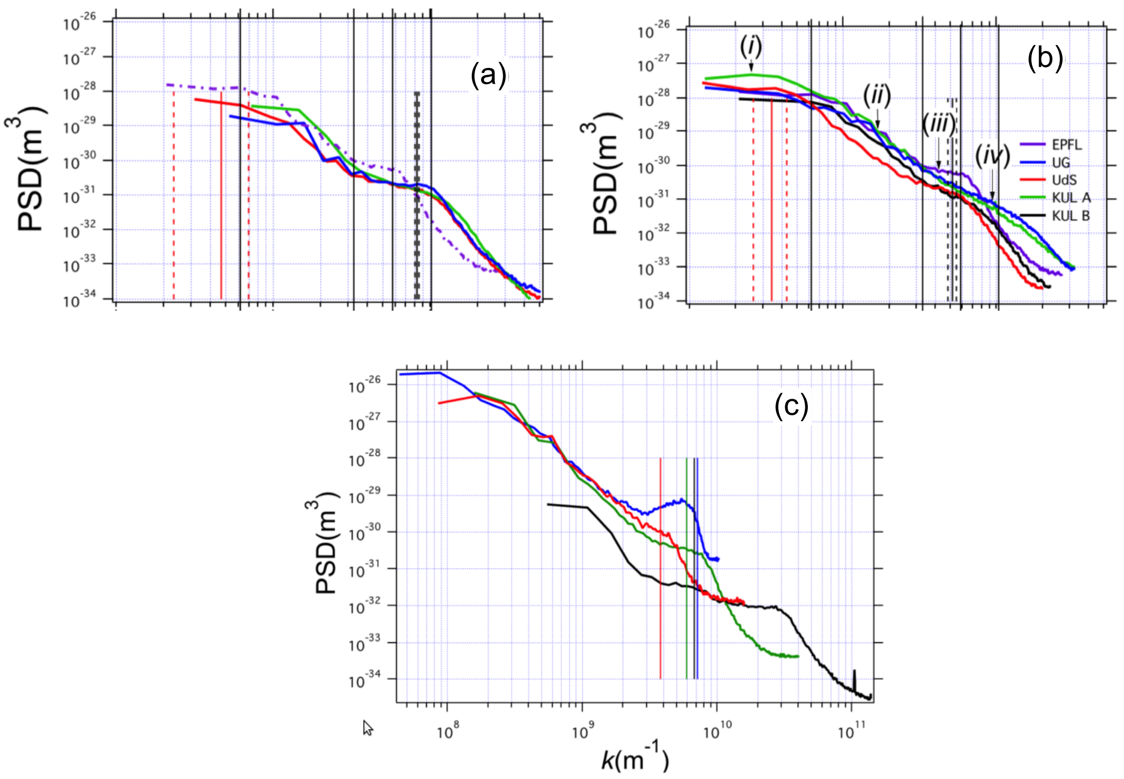

Figure S6. Representative figures from Biscarini *et al.* **[3]**. (a) PSD from STM data of homoligand (non-striped) nanoparticles. (b) PSD from STM data of 'striped' mixed-ligand nanoparticles recorded in 2012/2013. (c) PSD from STM data of 'striped' mixed-ligand nanoparticles recorded in 2004.

Figure S7(a) shows two functions (magenta and red) fitted to the blue data from Figure 3a) of Biscarini *et al.*. Vertical lines of the same colour show the position of the extracted characteristic frequency with 95% confidence bounds marked by dashed lines. The large variation in these fits show how the function is malleable enough to give a wide range of possible results biased by the initial fitting parameters.

The analysis of this curve in the original paper is, however, even more misleading than our re-analysis thus far would suggest. Only a small range of the data was fitted: the first plateau and some of the final noise were discarded [10]. Our attempt to recreate this fit (Figure S7(b) green curve) shows two characteristic frequencies marked by vertical lines. By the physical explanation presented in Biscarini *et al.* only one shoulder should be present in this region, yet two shoulders are fitted. They do not use the second, more prominent, shoulder; instead they use the first shoulder for their data. This shoulder is negligible in the context of the entire curve. No physical reasoning is ever given for the significant final shoulder. We also reiterate that **this data exclusion and choice of a central small shoulder rather than the dominant final is never mentioned in the text of Biscarini** *et al.* The frequency extracted from this small central shoulder agrees with the blue and green curves in Figure S6(b). In Figure S7(c) we overlay the fit from the supplementary information of Biscarini *et al.* (black curve) over the fits in S7(a) and (b). It is clear that this black curve almost totally obscures the green curve confirming that the green curve accurately recreates the fit of Biscarini *et al.*

It is therefore clear that the fitting is far from robust, and that sections of data were discarded to

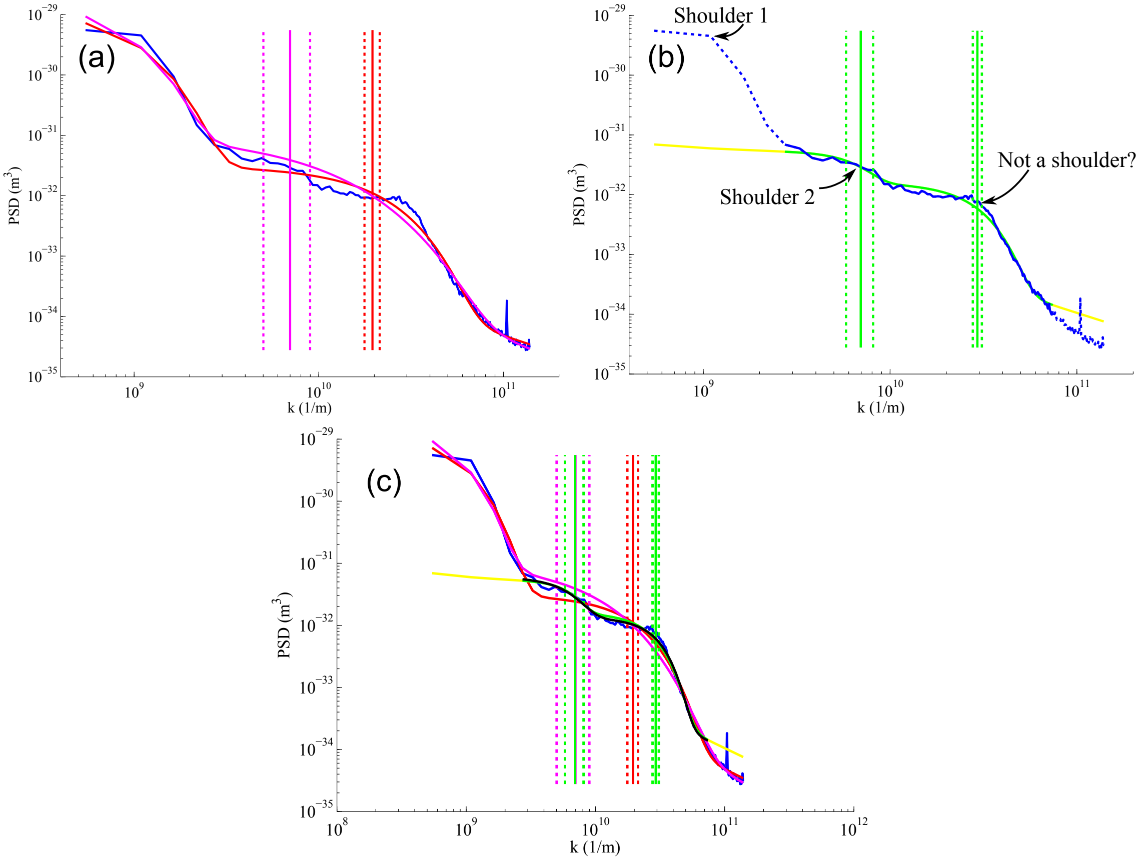

Figure S7. Fitting a 1D PSD from Biscarini *et al.* [**3**]. (a) Blue curve shows a 1D PSD from Black curve in Figure S6(c). Red and magenta curves show fits to this curve using the equation adopted for fitting by Biscarini and co-workers, with different seeded (i.e. initial) values. Vertical lines of the same colour show the characteristic frequency of the second shoulder returned from the fit. (b) Dashed regions of the blue curve show excluded sections of the blue curve from (a). The green curve shows a fit to this reduced data range. Vertical lines showing the characteristic frequencies of the first and second shoulder. As the first shoulder was discarded, Biscarini *et al.* use the first of these frequencies to define the second shoulder, even though their model expects only two shoulders and the next shoulder dominates. How these shoulders have been interpreted by Biscarini *et al.* is marked on the figure. The yellow curve is an extension of the green to show that the extended fit poorly fits the remaining excluded data. (c) Shows all data from (a) and (b) with the fit presented in the supplementary information of Biscarini *et al.* overlaid in black. The green curve is almost entirely obscured by the black demonstrating very similar fitting.

improve the consistency without this exclusion being mentioned in the text. We further note that all fits, including the green curve, were computed in MATLAB and gave warnings for poor convergence.

The above analysis was performed with Igor files provided by Fabio Biscarini (`.pxp` files) which are included in the directory `ACSNano_n_Langmuir2013`. The data named `lnk11` and `lnPSD11` and the corresponding fit have been imported into MATLAB and saved as the `.mat` file: `DataFromIgor.mat`. The function `FittingForFigure.m` fits for the full data using the equation from Biscarini *et al* [3] for two

seeded parameter sets using the MATLAB function `lsqcurvefit` for non-linear fitting. A further fit (as explained in the main text) is produced for a decreased range to attempt to match the fitting of Biscarini *et al.*

12 NMR spectroscopy

12.1 Further details on 1D spectroscopy of broad aryl peak

12.1.1 Further details for Janus nanoparticles

In the main text we describe problems with the linear model for the change in chemical shift presented in Equation 1 of Liu *et. al.* [2] for randomly mixed nanoparticles. The chemical shifts arise from the effects of localised magnetic fields generated in the aromatic rings of the DPT. For the Janus nanoparticles there is another fundamental problem with their approach. The authors assume that the position of the peak maximum, F, can be calculated as a superposition of two discrete chemical shifts, one originating from ligands in a bulk-like environment, and one coming from the interface between the two regions of the Janus particle.

This would be correct for two sharp, well separated lines that coalesce because of a fast mixing of the two components [11]. However, the two components clearly do not mix, since each ligand is fixed on the surface of the nanoparticles. In addition, the two lines are broadened due to an incomplete motional averaging of either anisotropic (orientation-dependent) interactions, such as dipole-dipole couplings or chemical shift anisotropies, or the presence of magnetic field gradients at the interface between the nanoparticles core and the organic shell due to different susceptibilities of the two. The two broad lines overlap significantly and the linewidths of the two regions are not necessarily identical (this would depend on the motion of the ligands in each domain as well as their packing). The qualitative trend of the expected peak position as a function of composition x_A should still be correctly described by the equation put forward by Liu *et al.*, but a correct quantitative description of the interface layer thickness t would only be coincidental. A quantitative estimate of t from an NMR data set may be possible by decomposing the line into a bulk component and a interface component, provided that the two lineshapes do not change as a function of x_A.

Aside from these fundamental limitations, the "rigorous" model presented by Liu *et. al.* [2] converges incorrectly at both concentration limits and leads to a clear trend in residuals. This comes from their treatment of the interaction band t being defined along the cross section of the particle rather than as a band of thickness $\tilde{t}$ on the surface of the particle (see Figure S8). This clearly has a strong effect at low or high concentrations where t should decrease in size, but remains constant in their treatment. Instead, the interaction area should be

$$A_{\text{int}} = 2\pi r_c \tilde{t} = 2\pi r \sin(\theta_c)\tilde{t}\,, \tag{1}$$

where r is the radius of the nanoparticle, and θ_c is the opening angle of the interface layer from the centre of the nanoparticle sphere. This form of the equation is not directly useful as we need to define $\sin(\theta_c)$ in terms of the height, h, of the spherical cap. From basic trigonometry

$$h = r(1-\cos(\theta_c)) \qquad \rightarrow \qquad cos(\theta_c) = 1 - \frac{h}{r}, \tag{2}$$

and using the identity $\cos^2(\theta) + \sin^2(\theta) = 1$:

$$\left(1-\frac{h}{r}\right)^2 + \sin^2(\theta_c) = 1 \tag{3}$$

$$\therefore \quad \sin(\theta_c) = \sqrt{1-\left(1-\frac{h}{r}\right)^2} = \sqrt{\frac{h}{r}\left(2-\frac{h}{r}\right)}\,. \tag{4}$$

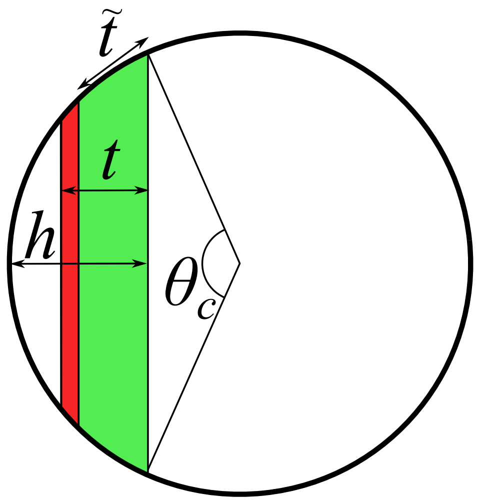

Figure S8. Area of the interaction stripe should be considered for an area thickness $\tilde{t}$ along the surface (green area) rather than a constant area thickness t along the cross section, otherwise the interaction area is significantly over estimated (red area) at high or low concentration.

Now combining Equations 1 and 4 we get:

$$A_{\text{int}} = 2\pi r\tilde{t}\sqrt{\frac{h}{r}\left(2-\frac{h}{r}\right)} = 4\pi r\tilde{t}\sqrt{x_A(1-x_A)}\,, \tag{5}$$

where $x_A = h/2r$ is the mole fraction of the thiol being detected.

In analogy to Liu *et. al.* [2], we take the equation for the area of a spherical cap to be the area taken for all molecules of this thiol,

$$A = 4\pi r^2 x_A\,. \tag{6}$$

Applying a constant chemical shift B for of thiols outside of the interaction band, covering an area $A - A_{\text{int}}$, and a chemical shift I for thiols within the interaction band , we arrive at a mean chemical shift of

$$F = \frac{(A - A_{\text{int}})B + A_{\text{int}}I}{A} \tag{7}$$

$$= B + \frac{\tilde{t}\sqrt{x_A(1-x_A)}(I-B)}{r x_A}\,. \tag{8}$$

This model, as mentioned in the main text, is not truly rigorous either because it is only valid for areas A where

$$A \geq A_{\text{int}}\,. \tag{9}$$

Notice that in the current definition, A_{int} only represents the fraction of the area within the interaction stripe that is covered by thiol A.

The data from Figure 3e in Liu *et. al.* [2], has been digitised using Engauge Digitizer (the digitised graphs are provided in the `NMR` folder of the supplementary information). Fitting the above model to the data using a least squares fitting algorithm in MATLAB, we arrive at a functional form of

$$F = 6.993 + \frac{.1464 \times \sqrt{x_A(1-x_A)}}{x_A} \tag{10}$$

where each data point is weighted by its vertical errorbar. See Figure S9 to compare the quality of the fit for the two models. Our model has an R^2 of .997 with no data points excluded, compared to the model of Liu *et. al.* with R^2 of .976 after arbitrarily excluding a point. This model is derived in full not because it has a direct impact on the evidence for striped morphologies for thiol capping layers, but instead to demonstrate careless data analysis and modelling that could easily be improved using the simple model described above.

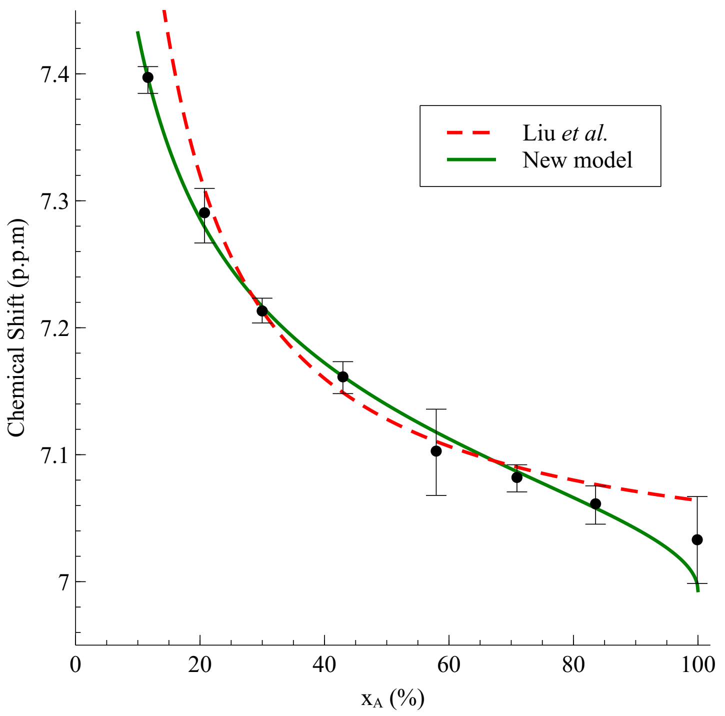

Figure S9. Digitised data for aryl peak chemical shift of Janus naoparticles from Liu *et. al.* [2]. The lines represent fits to the model from Liu *et. al.* (dashed) and our new model (solid).

12.1.2 Further details for patchy/striped nanoparticles

Liu *et. al.* [2] claim that the combination of their results they present can only be explained by the formation of stripe-like domains in the patchy nanoparticles. In the main text we suggested a different model that could also explain Liu *et al.*'s data, which we explain in more detail here. In this model the NMR data could also be caused by DPT molecules forming circular patches of well-defined size, with the phenyl rings stacked against each other. If the DPT/DDT ratio determines the number of patches, but not their size, the chemical shift of the NMR signal caused by the DPT would not significantly change over a relatively large concentration range. Essentially, from the point where enough DPT is present to form patches to the point where patches get so close to each other that they start interacting, the chemical shift would be constant. If the patches wwew circular or near-circular, and were big enough to accommodate a single DPT molecule in their centre, such a central DPT molecule may be able to rotate reasonably freely, which would cause a significant motional narrowing of its linewidth. However, rotation about the third axis would still be hindered, as this would require the whole nanoparticle to rotate, and hence some residual broadening is expected. We also note that ff the wall of the patches is caused by

stacked aryl rings, the central molecule would be in the plane of these rings, hence not experiencing an upfield shift (towards lower ppm values). In addition, the space on the nanoparticle's surface that is available to this molecule would be larger than the space available to DPT molecules that form the patch wall. Hence a larger reactivity could be expected. The narrow peak that is apparent in the broad aryl line could be explained by such an arrangement.

Moreover, the analysis of the ratios of the peaks presented in Table 2 would suggest that the patch wall consists of 16 to 26 DPT molecules, which would be sufficient to accommodate a single DPT molecule on the inside. Furthermore, due to the star-shaped arrangement of the aryl rings in the patch wall, the interface between DPT and DDT molecules would be fairly substantial (more substantial than in the case of a striped arrangement). Hence cross peaks in a 2D NOESY experiment would be expected, and their amplitude would even be larger than for a striped arrangement.

Although the current data cannot conclusively distinguish between such a patchy and a striped geometry of the ligand molecules, it is consistent with both interpretations. Hence, the NMR data presented does not conclusively show the presence (or absence) of stripe-like domains.

12.2 Discussion of narrow aryl peak

Liu *et. al.* [2] performed diffusion-ordered spectroscopy (DOSY) to measure the mobility of the broad aryl peak in comparison with the narrow aryl peak. The corresponding spectra are shown in the Supplement (Figure S14). Panels a and b show basically identical diffusion constants for the ligands that are causing the two peaks. This is to be expected, since the DOSY experiment essentially provides the mobility of the nanoparticles. Whether a ligand is covalently bound or non-covalently adsorbed on the surface cannot be assessed with this technique, since the distance travelled by an adsorbed molecule on the surface would be too small to induce an echo decay in a DOSY experiment: an adsorbed molecule would only travel a distance of ± 2 nm compared with the center of mass of a nanoparticle with a diameter of 4 nm, which is about three orders of magnitude too small a distance to be picked up with the employed gradient strengths [12]. That the molecule giving rise to the narrow peak is sticking to the surface of the nanoparticles in some way had already been established chemically, hence the DOSY data does not provide any additional information.

In addition, the analysis of the DOSY data is lacking an error estimate for the diffusion constants. The fits in Figure S14 a and b, using the Stejskal-Tanner equation, are not perfect, but the residuals are not very pronounced. Hence the assumption of a single diffusion constant appears to be a practical working model. However, the data for the mixture of nanoparticles and free ligand in panels c and d show very clear trends of the residuals. Fitting a single diffusion constant is misleading, and the obtained data does not represent a physical picture of the motion in the sample. An interpretation of the DOSY data in a two-dimensional way, where the chemical shift is correlated with a distribution of diffusion constants [13], would be a much more suitable method in this case. However, the information that could be obtained with respect to the ligand shell of the nanoparticles would still be very limited.

To demonstrate the increased reactivity of the molecules that cause the narrow aryl line, an experiment is presented where ligands were exchanged using aminoanthracene. The NMR spectra of the nanoparticles before and after this exchange were then compared (Figure 6). The two spectra were approximately scaled that the broad peaks had a comparable amplitude. However, the signal-to-noise ratio of the spectrum after the exchange is about an order of magnitude lower than before the exchange, and the lineshape of the broad line differs significantly between the two spectra. No experimental details were provided that would explain these differences. Assuming that the amount of sample was the same in both cases and that the spectra were recorded with an identical protocol, these data would imply that the aminoanthracene caused much more significant changes to the nanoparticles shell than just simply replacing the mobile aryl molecules, as suggested in the paper. A quantitative statement about the reactivity of the aryl molecules that cause the narrow peak in the original nanoparticles would require the spectrum after the exchange to be fully interpreted, including the sudden appearance of very narrow peaks and the change

of the shape of the broad line. In the current form, the two spectra are too different to conclusively show any of the claims made by the authors regarding the ligand reactivity.

12.3 Discussion of 2D NOESY data

Liu *et. al.* [2] also use 2D nuclear Overhauser exchange spectroscopy (NOESY) to analyse the ligand structure of gold nanoparticles. The authors base their arguments on the occurrence of cross-peaks between the alkyl peaks (occurring at a chemical shift of about 1 ppm) and the aryl peaks (with a chemical shift of about 7 ppm) in the NOESY data. Again the authors are selective in the information they analyse. Only the presence of a cross-peak or lack thereof is considered. However, the notable change in shape of the diagonal peaks is ignored. Such changes in diagonal peaks shapes can indicate a change in mobility.

When introducing the NOESY technique, the authors only state the dependence of cross-peaks on the distance between ligands. However, the cross-peak intensity is a product of the spectral density of the ligand motion at particular frequencies and, as correctly stated, the inverse of the distance between ligands to the sixth power [11]. As a consequence, a quantitative analysis of NOESY data necessarily requires to take the distance as well as the mobility into account. Certain ligands may show no cross-peak, even if they would be within the stated 0.4 nm from each other: the NOESY cross-peaks change their sign at a particular correlation time constant of the ligand motion, and when they do, the cross-peaks disappear.

Even if cross-peaks are not cancelled completely, a change in mobility, as caused by a size variation of the nanoparticles, can cause significant amplitude variation. If the cross-peak amplitude is close to the resolution limit, as appears to be the case in the presented data, a change of the diameter of the nanoparticles by a factor two, which is the size difference between the Janus nanoparticles that do not show cross-peaks and the other nanoparticles that do show cross peaks, could be the primary reason for the apparent absence of cross-peaks. Therefore a general statement that Janus particles can be distinguished by the absence of NOESY cross-peaks cannot be confirmed by looking at a single particle size only, especially since the other types of nanoparticles, which did show cross-peaks, had a significantly different size. Since this is the only information that was extracted from the NOESY data, these spectra do not seem to be useful for any conclusive statement without a more in-depth quantitative analysis. Such an analysis would have to consider not only the presence of cross-peaks, but also their amplitude and sign, as well as the mass and type of the nanoparticles.

Another shortcoming of the NOESY data is the apparently inconsistent use of contour levels between the spectra. This prevents a quantitative comparison of the cross-peak amplitudes from the data and an estimate of their lower limit. After all, even in the Janus nanoparticles there exists an interface between the two ligands that is not negligible for nanoparticles with a diameter of 2-3 nm, provided that the stated value of 0.4 nm for the layer thickness is of the right order of magnitude. Hence, although weaker, a cross-peak should become visible at some point for experimental conditions where cross-peak are not cancelled.

12.4 Section summary

In summary, the presented NMR data is not suitable to draw any conclusion about the structure of patchy/striped nanoparticles. This is not to say that NMR is an unsuitable tool for this kind of study, but the presented data and analysis do not support the conclusions that were drawn. The one exception is the authenticity and reproducibility of the narrow peak in the DPT-DDT nanoparticles, which was established chemically.

We would like to conclude by drawing an analogy, from an NMR point of view, between proteins and nanoparticles that are surface-coated with organic molecules. Despite their very different physical and chemical properties and different applications, the two may not be that different from an NMR point of

view. Their size, shape and mass can be comparable, and, hence, both may show similar mobility in a solvent.

While many nanoparticles cores are not directly accessible to study by NMR, an organic coating would be. If such a coating consists, as in the present case, of small molecules that can be easily distinguished by their chemical shift, many techniques that were developed for protein NMR may be applicable for the study of the surface coating of nanoparticles. Therefore, the choice of NMR by Liu *et. al.* [2] to probe the surface structure of nanoparticless is plausible, and NMR may in fact be able to answer many questions about the structure of organic molecules on the surface of nanoparticles in great detail.

NMR spectroscopy would not, however, be able to replace any of the imaging techniques, such as STM or TEM, for the study of nanoparticles. The information content of an NMR experiment is very different and in fact completely complementary to most of the techniques currently used routinely to study nanoparticles. It would appear to be worthwhile to apply the huge pool of methods and the experience in performing NMR experiments with large, low mobility proteins for the study of surface-coated nanoparticles. Collaborations between nanoparticle specialists and protein NMR groups, therefore, may prove to be very fruitful.

References

1. Raw data and analysis code on figshare. `http://dx.doi.org/10.6084/m9.figshare.882904`.

2. Liu X, Yu M, Kim H, Mameli M, Stellacci F (2012) Determination of monolayer-protected gold nanoparticle ligand-shell morphology using NMR. Nature communications 3: 1182.

3. Biscarini F, Ong QK, Albonetti C, Liscio F, Longobardi M, et al. (2013) Quantitative analysis of scanning tunneling microscopy images of mixed-ligand-functionalized nanoparticles. Langmuir 29: 13723–34.

4. Jackson AM, Myerson JW, Stellacci F (2004) Spontaneous assembly of subnanometre-ordered domains in the ligand shell of monolayer-protected nanoparticles. Nature materials 3: 330–6.

5. Stirling J, Woolley RAJ, Moriarty P (2013) Scanning probe image wizard: A toolbox for automated scanning probe microscopy data analysis. Review of Scientific Instruments 84: 113701.

6. Jackson AM, Hu Y, Silva PJ, Stellacci F (2006) From homoligand- to mixed-ligand- monolayer-protected metal nanoparticles: a scanning tunneling microscopy investigation. Journal of the American Chemical Society 128: 11135–49.

7. Ong QK, Reguera J, Silva PJ, Moglianetti M, Harkness K, et al. (2013) High-resolution scanning tunneling microscopy characterization of mixed monolayer protected gold nanoparticles. ACS nano 7: 8529–39.

8. Motulsky H, Ransnas L (1987) Fitting curves to data using nonlinear regression: a practical and nonmathematical review. FASEB J 1: 365–374.

9. Goldstein ML, Morris Sa, Yen GG (2004) Problems with fitting to the power-law distribution. The European Physical Journal B 41: 255–258.

10. Fabio Biscarini, Private communication, 2013.

11. Ernst R, Bodenhausen G, Wokaun A (1987) Principles of nuclear magnetic resonance in one and two dimensions. International series of monographs on chemistry. Oxford, England: Clarendon Press.

12. Callaghan P (1991) Principles of nuclear magnetic resonance microscopy. Oxford, England: Oxford University Press.

13. Thureau P, Thvand A, Ancian B, Escavabaja P, Armstrong GS, et al. (2005) Identification of two isomers from an organic mixture by double-stimulated-echo NMR and construction of the DOSY spectra by the regularized resolvent transform method. ChemPhysChem 6: 1510-1513.
